# Exploiting genetic variation to uncover rules of transcription factor binding and chromatin accessibility

**DOI:** 10.1038/s41467-018-03082-6

**Published:** 2018-02-22

**Authors:** Vivek Behera, Perry Evans, Carolyne J. Face, Nicole Hamagami, Laavanya Sankaranarayanan, Cheryl A. Keller, Belinda Giardine, Kai Tan, Ross C. Hardison, Junwei Shi, Gerd A. Blobel

**Affiliations:** 1 University of Pennsylvania, Philadelphia, PA 19104 USA; 2 Department of Biomedical and Health Informatics, Children’s Hospital of Philadelphia, Philadelphia, PA 19104 USA; 3 Children’s Hospital of Philadelphia, Philadelphia, PA 19104 USA; 4 Penn State University, University Park, PA 16802 USA

## Abstract

Single-nucleotide variants that underlie phenotypic variation can affect chromatin occupancy of transcription factors (TFs). To delineate determinants of in vivo TF binding and chromatin accessibility, we introduce an approach that compares ChIP-seq and DNase-seq data sets from genetically divergent murine erythroid cell lines. The impact of discriminatory single-nucleotide variants on TF ChIP signal enables definition at single base resolution of in vivo binding characteristics of nuclear factors GATA1, TAL1, and CTCF. We further develop a facile complementary approach to more deeply test the requirements of critical nucleotide positions for TF binding by combining CRISPR-Cas9-mediated mutagenesis with ChIP and targeted deep sequencing. Finally, we extend our analytical pipeline to identify nearby contextual DNA elements that modulate chromatin binding by these three TFs, and to define sequences that impact kb-scale chromatin accessibility. Combined, our approaches reveal insights into the genetic basis of TF occupancy and their interplay with chromatin features.

## Abstract (web summary)

Single nucleotide variants (SNVs) can affect chromatin occupancy by transcription factors (TF). Here the authors mine naturally occurring SNVs to probe *cis* elements and define contextual sequences that govern in vivo transcription factor chromatin occupancy and chromatin accessibility.

## Introduction

Genetic variation can modulate both rare and common disease susceptibility. Most genome-wide association study (GWAS) loci fall within non-coding regions of DNA and are particularly enriched in regions characterized by high chromatin accessibility and transcription factor (TF) occupancy^1–3^. Given the central role of TFs in regulating chromatin accessibility^4^ and transcription^5^, understanding the impact of genetic variation on TF binding may provide insights into the non-coding genetic components of development and disease.

In vitro binding assays and structural studies have been instrumental in identifying the DNA sequences that a TF can bind. In vivo, however, TFs typically occupy only a small proportion of their consensus binding sites. Hence, the presence of a TF consensus binding site has limited value in predicting actual TF occupancy^6,7^. In vivo TF binding is influenced by numerous features, including chromatin accessibility, histone modifications, and nearby chromatin occupancy of other TFs^8–10^. Given the marked heterogeneity in these attributes between different TF binding sites, it is perhaps unsurprising that methods, such as chromatin immunoprecipitation-sequencing (ChIP-seq) motif enrichment analysis, that compare binding between different genomic locations lack the sensitivity to identify contextual regulators of TF binding. Yet an understanding of these contextual regulatory elements is critical to interpreting the impact of non-coding genetic variants on TF occupancy.

Exploiting natural genetic variation in TF ChIP-seq data sets provides a means to pinpoint nucleotides critical for direct TF binding as well as to identify contextual regulators that function outside the core binding motifs. It allows direct comparisons between different alleles or individuals that have similar if not identical contextual *cis* attributes and *trans*-acting environments and yet contain a small number of discriminatory genetic polymorphisms. For example, comparative ChIP-seq and DNase-seq between different strains of mice has identified sequence variants in cofactor motifs that affect PU.1 and C/EBPɑ binding^11,12^. Others have identified allelic imbalance in TF binding by gathering ChIP-seq or DNase-seq data in hybrid mouse strains^13^ or in human lymphoblastoid cell lines^14,15^ and patient tissues^16^ that are replete with sequence variants.

A rich source of natural genetic variation can be found among the extensively characterized ENCODE cell lines^1^. Especially useful are lines from distinct genetic backgrounds but representing the same cell types with highly similar transcription profiles. For example, the murine G1E-ER4 and murine erythroleukemia (MEL) erythroid cell lines are derived from different genetic backgrounds but are both similar to primary murine erythroblasts; each of these lines has multiple available RNA-seq, DNase-seq, and ChIP-seq data sets. While the majority of ENCODE cell lines lack whole-genome sequencing (WGS), we found that input ChIP-seq data can be exploited to sensitively and accurately identify sites of natural genetic variation. We subsequently use this variation as a tool to better understand the in vivo binding determinants of the major erythroid TFs GATA1 and TAL1, and the chromatin architectural protein CTCF. Specifically, our approach defines sequence motifs that these TFs contact within chromatin, and identifies nearby contextual DNA elements and their associated proteins that positively or negatively impact TF chromatin occupancy. We then complement this approach by generating a spectrum of mutations in select regions followed by ChIP and massively parallel sequencing. Moreover, we identify genetic variants that alter chromatin accessibility as a result of TF binding site disruption. Collectively, we describe a practical approach to mine data sets for both natural genetic variation and TF binding profiles in order to define how TFs bind and regulate chromatin.

## Results

### Exploiting ChIP-seq data to uncover genetic variation

**Fig. 1:**
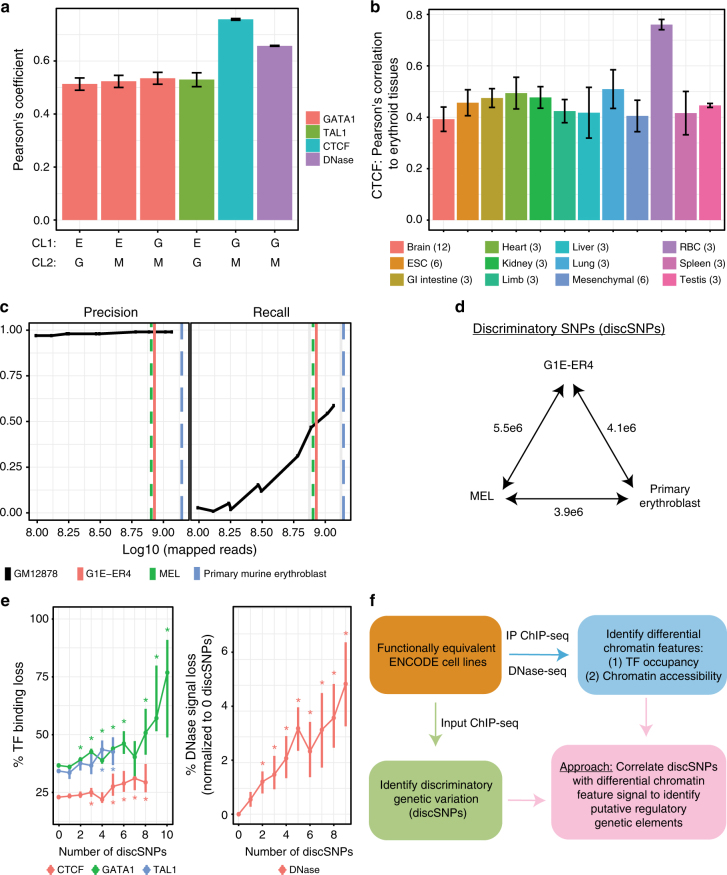
Control ChIP-seq data reveals extensive genetic variation between functionally equivalent ENCODE cell lines. **a** Pearson's correlation coefficient (PCC) of TF binding and DNase hypersensitivity profiles between pairs of erythroid cell lines (E = erythroblast, G = G1E-ER4, M = MEL) at commonly called peaks. PCC ± 95% CI. **b** PCC of CTCF binding between indicated tissues and erythroid tissues (G1E, G1E-ER4, MEL). Mean ± SEM, number of comparisons listed in figure. **c** Precision and recall of using input ChIP-seq data in GM12878 cells to identify homozygous variants relative to the hg19 reference genome. Vertical lines denote the number of input ChIP-seq reads available for the murine erythroid cell lines. **d** Number of discriminatory SNP (discSNP) variants between each pair of erythroid cell lines. **e** Median percent signal loss (relative to stronger binding signal) at TF peaks or DNase hypersensitivity (DHS) peaks between erythroid cell lines, separated by the number of discSNPs located within the TF/DHS peak. TF binding loss % with 0 discSNPs reflects the background level of variation in TF peak intensities between cell lines despite identical underlying DNA sequences. DNase percentages are normalized to the 0 discSNP data point within peaks of identical length. *Wilcoxon's *p* < 0.05 for comparison to peaks lacking discSNPs. Vertical bars represent 95% confidence intervals by bootstrapping. **f** Schematic of the overall analysis approach that uses genetic variants to probe determinants of TF binding and chromatin accessibility

G1E-ER4 and MEL are cell lines commonly used for studying erythroid differentiation. These cells can be induced to differentiate, upon which broadly similar sets of genes are induced or repressed when compared to primary murine erythroblasts^17,18^ (Spearman's *r* = 0.56–0.72, Supplementary Fig. 1a, b, c). Rich genome-wide data sets on chromatin features are available for G1E-ER4, MEL, and primary erythroblasts through ENCODE^19^. A comparison between these cell types revealed strong positive correlations in ChIP-seq peak binding intensities of two major hematopoietic TFs GATA1 and TAL1, as well as in DNase I accessibility, reflecting similar regulatory landscapes (Fig. 1a). CTCF, a ubiquitously expressed factor with roles in chromatin architecture, also exhibited highly correlated peak binding intensities among red cell types (Fig. 1a). Cross-comparisons of CTCF-binding profiles between tissues showed remarkably correlated binding patterns, likely reflecting the tissue-invariant fraction of CTCF-binding sites. Notably, erythroid tissues are more correlated in CTCF-binding profiles to each other than to other tissues (Fig. 1b), consistent with patterns comprising both tissue-restricted and tissue-invariant CTCF occupied sites.

Despite their phenotypic similarity, each of the three sources of erythroid cells originated from a different inbred mouse strain^20,21^ leading to the expectation that they harbor discriminatory genetic variants that might impact chromatin characteristics. Since the cell lines lack WGS data, we mined ChIP-seq unprecipitated “input” data sets available from ENCODE (Supplementary Data 1) to identify such variants relative to the C57BL/6J reference genome build^22^ (Supplementary Fig. 2a). For primary erythroblasts, since relatively few ChIP-seq data sets are accompanied by sequencing of “input” material, we additionally used the background reads from the immunoprecipitated material (excluding GATA1/TAL1/CTCF ChIP data) for variant identification. We adapted the GATK HaplotypeCaller tool^22^ for variant identification, applying stringent criteria (detailed in Methods) for assigning mutation zygosity. We benchmarked the sensitivity and accuracy of this approach by first using ENCODE input ChIP-seq data to call genetic variants in the GM12878 human lymphoblastoid cell line relative to the Hg19 genome assembly, and then by comparing called variants to the recently sequenced GM12878 genome^23^. Homozygous genetic variation was identified with nearly 100% precision (% of identified variants that are true variants) and >50% recall (% of true variants identified) (Fig. 1c). Since variant identification for the GM12878 cell line benefits from an unusually large number of input ChIP-seq files available from ENCODE (57 files resulting in 1.4 billion unique reads), we examined whether fewer input data sets would permit variant identification. We found that randomly downsampling the number of GM12878 reads to the numbers available for the murine erythroid cell lines still identified homozygous genetic variation with nearly 100% precision and ~50% recall (Fig. 1c). We conclude that input ChIP-seq files enable stringent variant identification comprising at least half of existing genome-wide variation in ENCODE murine erythroid cell lines.

Applying these methods to the three murine erythroid cell models identified 1.7–2.6 million homozygous variants (relative to the reference mm9 genome) from input ChIP-seq data alone. Additionally, the G1E-ER4 and MEL cell lines are derived from mouse strains that have been sequenced by the Sanger Institute Mouse Genomes Project^24^. Merging the variant calls from input ChIP-seq data and those made by the Sanger Institute confirms that we can use input ChIP-seq files alone to detect 41–47% of total genetic variation. Of note, 4–6% of variants present in the erythroid cell lines are not found in their parent mouse strains (Supplementary Fig. 2b), suggesting that they arose during culture. In total, these data reveal that each pair of cell line genomes is discriminated by 3.9–5.5 million single-nucleotide polymorphisms (SNPs) (discSNPs) (Fig. 1d). Together, identification of SNPs directly from ChIP-seq data and from WGS data (when available) serve as complementary approaches that make discriminatory variant analysis possible even in cell lines lacking WGS data.

Importantly, we found a nearly monotonic increase in relative loss (read count intensity in weaker allele relative to stronger allele) of TF binding (GATA1, TAL1, or CTCF) or chromatin accessibility as the number of discSNPs located within the peak increases (Fig. 1e). These findings suggest that a subset of discSNPs alters chromatin features between these murine erythroid cell lines. We thus sought to exploit these discSNPs in a comparative analysis of existing ChIP-seq and DNase-seq data in order to identify genetic determinants of TF occupancy and chromatin accessibility in erythroid cells (Fig. 1f).

### Genetic variants alter GATA1 binding and gene expression

**Fig. 2:**
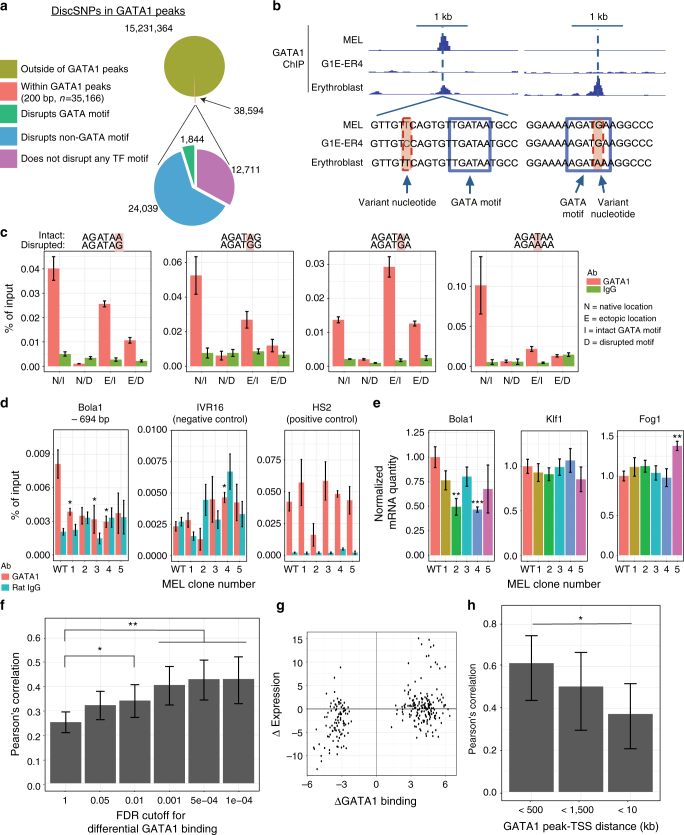
Isolated genetic variants within GATA1 peaks co-occur with dramatic changes in GATA1 binding and nearby transcription. **a** Summary of discSNP pairs in GATA1 peaks (200 bp, *n* = 35,166). **b** GATA1 ChIP-seq intensity tracks (input and library-size-normalized, identical *y*-axis scales) reveal dramatic changes in GATA1 binding associated with single-nucleotide variants adjacent to (chr3: 66,455,298 - 66,457,298) or within (chr19: 46,129,935 - 46,131,935) a GATA1 motif (no other discSNPs within 1 kb). Dotted vertical line indicates discSNP position. **c** GATA1 ChIP-qPCR at native (N) loci in either G1E-ER4 or MEL cells where one cell line contains an intact (I) GATA1 motif and the other cell line has a disrupted (D) motif. A 200-bp region centered on these intact/disrupted motifs was barcoded and cloned into ectopic (E) locations in G1E-ER4 cells and GATA1 ChIP-qPCR was performed at these sites. Mean ± SEM, *n* = 3. **d** GATA1 ChIP-qPCR and **e** RT-qPCR in 5 MEL clones edited at a control locus (WT) or at the *Bola1*-proximal GATA1 peak. For **d**–**e**, mean ± SEM, *n* = 3. **f** For discSNPs found in TSS-proximal GATA1 peaks, Pearson's correlation coefficients between delta GATA1 binding and delta transcription at a range of FDR cutoffs for differential binding. **g** Scatterplot of delta GATA1 binding vs. delta transcription at an FDR cutoff of 1e−4, PCC = 0.43. **h** Pearson's correlation coefficients between delta GATA1 binding and delta transcription at a range of cutoffs for distance between the GATA1 peak and nearby TSS. For **f**–**h**, error bars are 95% confidence intervals, *p = 0.03, **p < 0.001 (Fisher’s Z-transform)

Upon intersecting the ~15 million discSNPs in erythroid cells with GATA1 chromatin occupancy data in these cells, we found 38,594 discSNPs that fall within regions identified as GATA1 peaks in at least one erythroid cell line (Fig. 2a). We hypothesized that isolated genetic variants (no other variants within 1 kb) within GATA1 peaks may either directly or indirectly interfere with GATA1 chromatin occupancy. Indeed, we uncover single-nucleotide discSNPs associated with dramatic changes in GATA1 binding that reside within or immediately adjacent to the GATA motif (Fig. 2b). GATA1 ChIP-quantitative PCR (ChIP-qPCR) in differentiated G1E-ER4 and MEL cells confirms complete loss of GATA1 binding at several sites containing a single isolated discSNP (Supplementary Fig. 3a). In some instances, these variants are associated with large corresponding changes in proximal gene transcription (Supplementary Fig. 3b).

For four discSNPs falling within GATA1 motifs, we tested whether they were causal in altering GATA1 binding. To this end, we cloned 200 bp regions encompassing the discSNPs into a retroviral vector that randomly inserts the cloned element along with a barcode into the G1E-ER4 genome. GATA1 ChIP-qPCR at these ectopic sites recapitulated the allele-specific differences in binding seen at endogenous loci (Fig. 2c). However, the effect size of the SNP on binding was smaller at the ectopic sites, possibly due to a tendency of retrovirus to integrate into open chromatin, whereas the endogenous sites devoid of GATA1 binding can lose chromatin accessibility (see below).

In order to validate causality of a disruptive SNP on transcription we initially attempted homologous recombination (HR)-driven CRISPR-Cas9-driven point mutagenesis. However, the efficiency of this process in MEL cells was too low, prompting us to instead generate via CRISPR-Cas9 small deletions at a test location harboring a discSNP and measure the effect on GATA1 binding and local gene transcription. The *Bola1* gene transcription start site (TSS) is 700 bp downstream of a GATA1 peak that contains a single G1E-ER4 discSNP predicted to disrupt a GATA1 motif. This genetic change is associated with significantly reduced GATA1 binding (*q* = 0.024) and *Bola1* transcription (*q* = 0.005) in G1E-ER4 cells (Supplementary Fig. 3c). We found that multiple MEL clones containing bi-allelic deletions (4–22 bp) of the discSNP region (Supplementary Fig. 3d) exhibit minimal GATA1 binding and diminished transcription (Fig. 2d, e). Together, these findings support that discSNPs within GATA1 motifs can directly impact GATA1 chromatin occupancy and nearby gene transcription. Moreover, a small deletion adjacent to but not overlapping the GATA1 motif, as is the case for clone 2, can have equally  detrimental effects as deletions of the GATA1 motif itself, highlighting the importance of sequence context in chromatin occupancy (see below).

GATA1 has been previously shown to mediate widespread gene regulation during terminal erythroid differentiation^17,25,26^. We thus tested whether discSNPs that alter GATA1 binding impact nearby gene transcription on a genome-wide scale. In GATA1 peaks containing at least one discSNP, there is a positive correlation between changes in GATA1 binding and in nearby gene transcription; this correlation becomes stronger as the false discovery rate (FDR) threshold for differential binding (Benjamini–Hochberg corrected, using DiffBind^27^) is increased (at FDR 1e−4, Pearson's correlation coefficient = 0.43, *p* = 8e−14) (Fig. 2f, g). GATA1 binding and transcriptional changes become less correlated with increasing GATA1 peak-to-TSS distances, suggesting that GATA1 binding is more likely to regulate transcription of the nearest gene at shorter distances (Fig. 2h). These associations might underestimate the impact of discSNPs on transcription since proximity is an imperfect means of pairing regulatory elements with their target genes. Together, these data suggest that discSNPs can causally underlie changes in GATA1 chromatin occupancy and transcriptional regulation.

### In vivo GATA1 binding determinants assessed at single base resolution

**Fig. 3:**
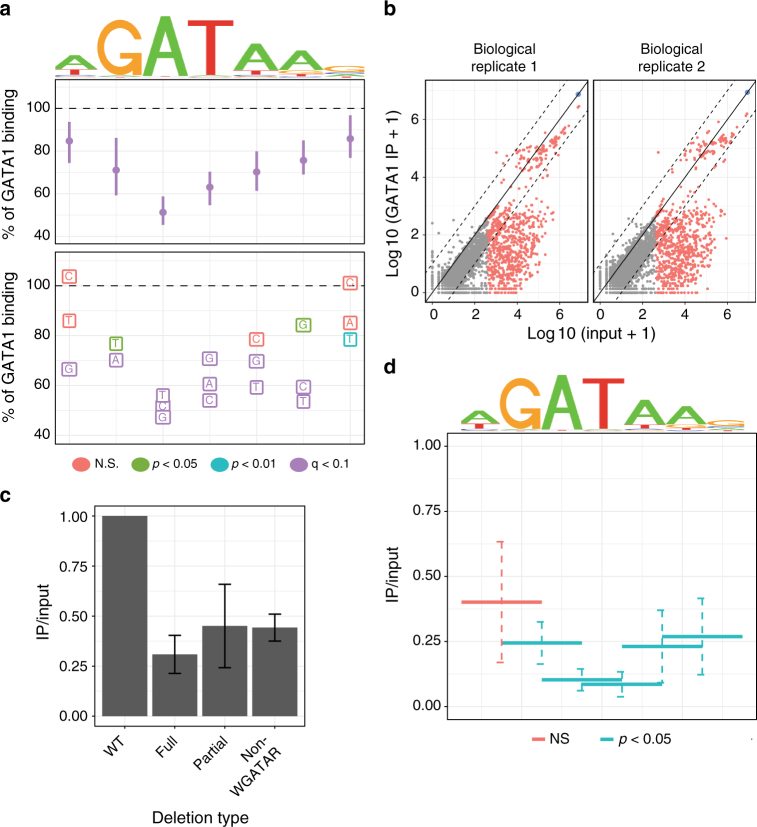
Genetic variation reveals sequences that directly regulate GATA1 chromatin occupancy. **a** Sequence logo reflecting canonical GATA1 motif and the percent impact on GATA1 binding intensity associated with discSNPs at various positions and to particular alternative nucleotides. Median ± 95% confidence intervals by bootstrapping. **b** Normalized read count in sequenced ChIP input vs GATA1 IP for deletion-containing alleles of the *Bola1*-proximal GATA1 peak in MEL cells. Gray points indicate deletions removed due to low Input read counts, red points indicate deletions above the read count threshold, and blue point indicates wild-type (non-deleted) MEL allele. Solid line indicates 1:1 normalized IP enrichment, and dashed lines indicate 10-fold changes in enrichment. **c** Aggregate effects on GATA1 IP enrichment of either full or partial deletions of the WGATAR motif or of deletions not overlapping this motif, mean ± SEM. **d** Aggregate effects of 1-bp or 2-bp deletions within a 2-bp sliding window across the GATA1 motif on GATA1 IP enrichment, mean ± SEM

We examined the genome-wide impact of discSNPs on GATA1 binding as an unbiased means to define in vivo genetic determinants of GATA1 chromatin occupancy. We used the MEME suite^28^ and CIS-BP motif database^29^ to identify discSNP pairs that fall within a TF motif and are predicted to disrupt binding to that motif (detailed in Methods). We found that 67% of all discSNPs in GATA1 peaks disrupt a TF motif, with the majority being non-GATA motifs (Fig. 2a). GATA1 is the primary GATA factor in differentiated erythroid cells^30^ and it binds to sequences described in the CIS-BP database as GATA1/2/4/6 motifs that we collectively treat as the GATA family motif (WGATAR) (Supplementary Fig. 4a, b). We use the 1844 discSNPs disrupting a GATA motif and measure their impact on GATA1 chromatin occupancy. Our analysis compares normalized GATA1 signal intensity in the allele with a disrupted motif relative to the allele with an intact motif (% of GATA1 binding) and uses non-parametric statistical methods and permutation-based corrections for multiple hypothesis testing to identify nucleotides that significantly contribute to GATA1 binding (detailed in Methods). Indeed, we found that disruption of any nucleotide in the WGATAR consensus sequence results in a statistically significant loss of GATA1 binding (Fig. 3a). Importantly, although the relative expression levels of signature erythroid genes^18^ suggest that primary erythroblasts are slightly more differentiated than G1E-ER4 and MEL (Supplementary Fig. 1b, c), the effects of a mutated GATA motif on GATA1 binding are consistent regardless of which pair of erythroid cell lines is compared (Supplementary Fig. 4c). These data indicate that differential GATA1 binding associated with genetic variation does not reflect differences in *trans* factor environment.

Evidence from PCR-mediated site selection, electrophoretic mobility shift assay, and ChIP-seq experiments suggests that the central GATA nucleotides are required for in vitro GATA1 binding^31,32^ and are found with near 100% frequency in the in vivo occupied GATA1 binding motif^7,33^. We found, however, that these core motif nucleotides contribute unequally to GATA1 binding in vivo despite near 100% occurrence at GATA1-bound sites. Additionally, while in vitro SELEX experiments suggest that the G is more essential to binding than the first A, we found that disruption of the G results in a milder effect on in vivo GATA1 binding (29% ChIP signal loss) than disruption of the A (49% ChIP signal loss, Wilcoxon's *p* = 0.004). These data suggest that in vivo TF chromatin interactions are not identical to in vitro TF DNA oligonucleotide interactions and are modulated by factor or chromatin context. Additionally, we identify nucleotide substitutions that strongly impair GATA1 binding in a manner dependent on the type of substitution (Fig. 3a). Together, these results provide a framework for understanding the quantitative impact of any substitution within the GATA1 motif on in vivo GATA1 chromatin occupancy.

In order to directly test the causality of select discSNPs on GATA1 DNA binding, we attempted to carry out HR-directed CRISPR-Cas9-mediated mutagenesis but found this process to be of very low efficiency in our erythroid cell lines. Instead, to experimentally test endogenous GATA1 binding preferences, we developed an approach that combines CRISPR-Cas9, conventional GATA1 ChIP, and targeted deep sequencing to interrogate GATA1 binding determinants in a high-throughput and high-resolution manner. As before, we targeted the *Bola1*-proximal GATA1 peak in MEL cells using a guide RNA (gRNA) designed to cleave within the GATA consensus motif. Deep sequencing of genomic DNA from edited MEL cells reveals a diverse range of deletions averaging 26 bp long; 50% of these completely delete the WGATAR sequence and 41% partially delete this sequence (Supplementary Fig. 4d, e). We performed GATA1 ChIP on these cells followed by targeted deep sequencing of both ChIP input and IP material, and found a large number of deletions that are depleted in the IP library relative to their starting abundance (Fig. 3b). GATA1 binding is substantially reduced by both full and partial deletions of the GATA1 motif (Fig. 3c). Surprisingly, GATA1 binding is similarly diminished by deletions adjacent to but not containing the GATA1 motif, implicating nearby non-WGATAR sequences in regulating GATA1 binding (see below). We next used a 2-bp sliding window to analyze the effects of 1-bp and 2-bp long deletions within the GATA1 WGATAR motif. We found similar nucleotide sensitivities (Fig. 3d) as determined by genome-wide discSNPs (Fig. 3a), suggesting that discSNPs within the GATA1 motif reveal causal nucleotide determinants of in vivo GATA1 chromatin occupancy. Together, these discSNP-mediated and CRISPR-Cas9-mediated approaches enable definition of in vivo GATA1 DNA-binding determinants at base pair level resolution.

### Nearby contextual sequences regulate GATA1 binding

**Fig. 4:**
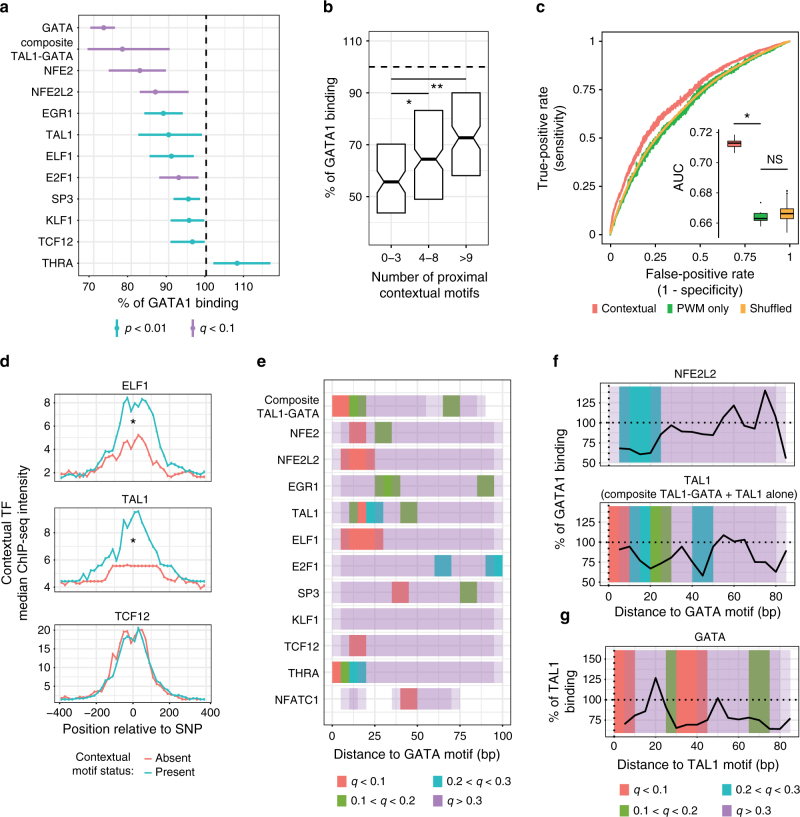
Genetic variation in nearby contextual sequences regulates GATA1 binding. **a** TFs whose motifs significantly alter GATA1 binding (% impact, median ± 95% CI) when disrupted by a discSNP. Vertical line indicates no effect, color indicates significance level. **b** DiscSNPs that directly disrupt a GATA motif have variable impacts on GATA1 binding depending on the number of positive co-regulatory motifs (from **a**) found within 100 bp of the discSNP. Wilcoxon's *p = 0.02, **p < 0.0004. Boxplot center is median, hinges are 25 and 75% percentiles. **c** Receiver operating characteristic curve (ROC) curves comparing logistic regression models that predict GATA1 binding based on either the GATA1 motif alone (PWM only), a combination of the GATA1 motif and nearby contextual regulatory motifs (contextual), or a control combination of the GATA1 motif and scrambled versions of the contextual motifs (shuffled). ROC curves are shown as median of 10 cross-validation runs. Area under the curve (AUC) is represented in the sub-panel as a boxplot of the 10 cross-validation runs, DeLong’s paired test for two correlated ROC curves: *p = 9e−13. Boxplot center is median, hinges are 25 and 75% percentiles, whisker is no >1.5*IQR. **d** Median ChIP-seq intensity of three TFs at their corresponding motifs in either an intact state or disrupted by a discSNP within a GATA1 peak. Position is shown relative to the discSNP position (400 bp upstream to 400 bp downstream). Significant differential binding (*) was assessed by a BH-corrected t test in the −200 to +200 region (TAL1: *q* = 0.008, ELF1: *q* = 0.008, TCF12: *q* = 0.25). **e** Sliding bins (10-bp wide, overlapping by 5 bp) test the impact of contextual motif disruption on proximal GATA1 binding as a function of relative distance to the nearest GATA1 motif. Colors represent significance in altering binding, permutation-adjusted for multiple hypothesis testing. **f** Sliding window medians of the impact of NFE2L2 and TAL1 motif disruption on GATA1 binding as a function of distance to motif. **g** Sliding window medians of the impact of GATA1 motif disruption on TAL1 binding as a function of distance to the nearest TAL1 motif

We next turned our focus to the 24,039 discSNPs located in GATA1 peaks that alter a non-GATA motif as a means for identifying potential genetic and protein regulators of GATA1 chromatin occupancy. While both in vitro^34,35^ and in vivo^36–38^ studies have identified proteins likely to interact with GATA1 in transcriptional regulation, little is known about how these proteins impact GATA1 chromatin occupancy on a genome-wide scale. Notably, GATA1 chromatin binding can be collaboratively regulated by both DNA-binding proteins such as KLF1^39^ and by non-DNA-binding proteins such as FOG1^40–42^ and LDB1^43^, although only FOG1 has been described to impact GATA1 binding on a genome-wide scale. Of note, it has not been established whether DNA binding by other TFs that can physically interact with GATA1, such as TAL1 or NFE2, impacts GATA1 binding in a similarly collaborative fashion. In fact, analysis of discSNPs disrupting non-GATA TF motifs reveals multiple DNA sequences (NFE2, NFE2L2, E2F1) with significant (*q* < 0.1) positive contributions to GATA1 binding (Fig. 4a). Additional motifs, including those associated with proteins previously shown to interact with GATA1, have less significant median effects on GATA1 binding. DiscSNPs that disrupt the KLF1 motif, for example, have in some cases dramatic effects on GATA1 binding but in the majority of cases relatively little effect, likely due to the low genome-wide co-localization of KLF1 and GATA1^44^ and the impact of contextual constraints such as motif spacing and orientation. Strikingly, we found that the disruptive effect of a discSNPs within a GATA motif deteriorates as the number of adjacent TF binding sites (non-GATA motifs that promote GATA1 binding) increases (Fig. 4b). We also found that discSNPs disrupting either a GATA motif or a proximal contextual motif have markedly stronger effects in ablating GATA1 binding as the predicted binding affinity (based on MEME score) of the disrupted motif increases (Supplementary Fig. 5a). These data directly implicate proximal contextual motifs as a mechanism to buffer in vivo GATA1 binding against motif mutations that would severely impact binding in vitro.

Despite extensive in vitro and in vivo studies that identified motifs for GATA1 and other TFs, prediction of genome-wide binding of these factors has been limited by a lack of consideration of genomic context. While predictions have been substantially improved by the integration of chromatin features such as accessibility^8,16^ or histone marks^9^, these data are not always available, and it remains unclear how to accurately predict TF binding to a genomic region from DNA sequence alone. To this end, we hypothesized that nearby genetic sequences that regulate GATA1 binding may better predict whether GATA1 binds to its canonical motif. We trained a logistic regression model for the genome-wide prediction of GATA1 binding that incorporates as features both the GATA motif match score and match scores for each contextual motif found within 100 bp of the GATA motif. A model that considers not only GATA motif matching but nearby motif matches to seven other TFs (HIVEP1, KLF1, NFE2, NFE2L2, TAL1, composite GATA-TAL1, TCF12) significantly outperforms (*p* = 9e−13) both a model that only considers GATA and a control model that considers GATA along with randomly shuffled versions of these seven motifs (Fig. 4c). At an FDR of 0.3, this results in an increase from 34 to 57% of true GATA1 peaks that are correctly identified when considering potential binding of nearby contextual factors (Supplementary Fig. 5b). We were limited in making this comparison between prediction models at FDR values <0.25 since the GATA motif-only model has a sensitivity of 0% at these values. These data illustrate the need to consider nearby contextual regulatory sequences when evaluating TF binding.

We limited our analysis to DNA motifs whose corresponding TFs are expressed in erythroid cells (Supplementary Fig. 5c). However, we found that a number of these motifs correspond to families of TFs where family members bind highly similar sequences (Supplementary Fig. 5d), and thus the TF protein binding these sequences cannot be unambiguously assigned. To better understand the proteins that regulate GATA1 binding, we exploited existing erythroid ChIP-seq data for TAL1, ELF1, and TCF12 to test whether binding by these proteins reflects the impact of discSNPs on their corresponding DNA motifs. DiscSNPs that disrupt motifs for TAL1 and ELF1 in GATA1 peaks are associated with concomitant loss of TAL1 and ELF1 protein at these locations, while disruption of TCF12 motifs have relatively little effect on TCF12 occupancy (Fig. 4d). This implicates TAL1 and ELF1 but not TCF12 as candidate factors that promote GATA1 binding on a genome-wide scale, while the effects seen by TCF12 likely instead result from E2A, an obligate heterodimer of TAL1^45^ that binds a highly similar TF motif to TCF12 (Supplementary Fig. 5d).

TF motif spacing has been previously shown to provide strict constraints on collaborative binding^46^ and enhancer function^47,48^. For example, GATA1 has been shown to frequently bind adjacent GATA and E-box motifs spaced 8–10 bp apart^43,49^, and reporter assays find this particular spacing to be critical for the activity^50^. Thus, we examined the effect of distance between non-GATA motifs that affect GATA1 occupancy that are altered by discSNPs and the nearest GATA motif. While for some motifs the data were too sparse for this analysis, we uncovered motifs that regulate GATA1 binding predominantly at short distances (<30 bp, TCF12, THRA), or long distances (30–50 bp, SP3, E2F1) (Fig. 4e, f). Surprisingly, several motifs, including the E-box commonly bound by TAL1, regulate GATA1 binding at a wide range of distances (0–50 bp) that have not have been predicted by either motif enrichment analysis or enhancer reporter assays. Similarly, analysis of discSNP effects on TAL1 ChIP-seq signal in G1E-ER4 vs. primary erythroblasts shows that GATA1 motif mutations impact TAL1 binding at distances as great as 75 bp (Fig. 4g), supporting previously unappreciated long-range collaborative binding between the master erythroid factors GATA1 and TAL1. These data demonstrate that natural genetic variation is a sensitive method for uncovering contextual rules that govern in vivo TF chromatin occupancy.

### Contextual motifs influence CTCF chromatin occupancy

**Fig. 5:**
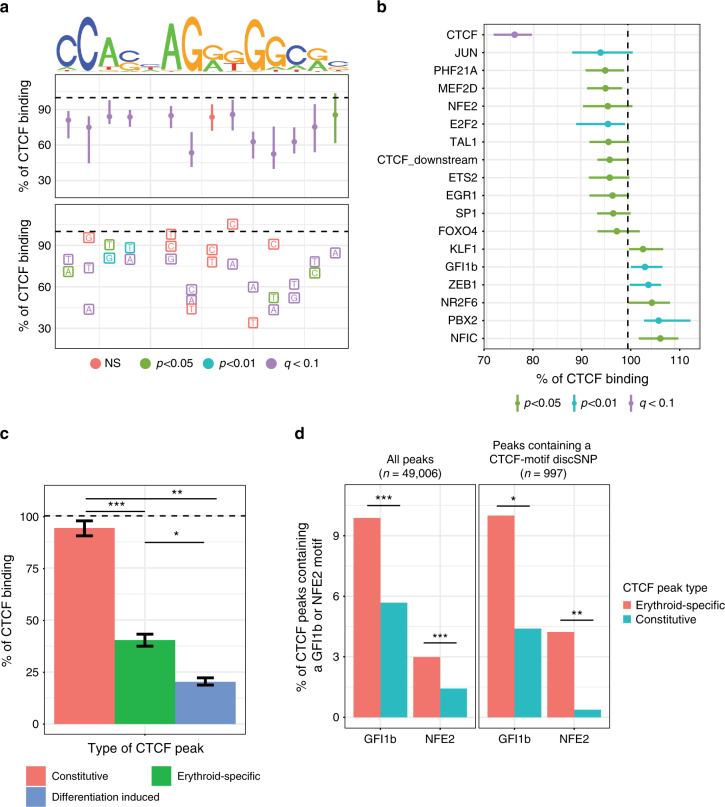
Genetic variation between erythroid cell lines reveals genetic regulatory sequences directing CTCF chromatin occupancy. **a** Sequence logo reflecting existing CTCF factor motif information and the percent impact on CTCF binding intensity (median ±  95% CI) associated with mutations at various positions and to particular alternative nucleotides. **b** TFs whose motifs significantly alter CTCF binding (% impact, median ± 95% CI) when disrupted by a discSNP. Vertical line indicates no effect, and color indicates significance level. **c** The effect of discSNPs that disrupt CTCF motifs on CTCF binding at constitutive, erythroid-specific, or erythroid differentiation-induced CTCF peaks. Percent impact, median ± 95% CI. * Wilcoxon's *p* = 1e−6, **p = 1.7e−14, ***p = 4.7e−39. **d** GFI1b and NFE2 motifs are enriched in erythroid-specific CTCF peaks (foreground) relative to constitutive CTCF peaks (background) both when considering all CTCF peaks and the subset that contain a CTCF motif discSNP as in Fig. 5c. Benjamini–Hochberg *q*-values: *q = 0.02, **q = 0.001, ***q = 0.0000

Analysis of genetic variation not only allows us to understand how erythroid-specific factors bind chromatin but also how ubiquitously expressed TFs, such as CTCF, bind chromatin in a tissue-specific manner. While CTCF binds many locations in all cell types, a fraction of its binding sites is dynamic and lineage-specific^51–53^, including in erythroid cells (Fig. 1b). We hypothesized that analysis of discSNPs that disrupt either constitutive or erythroid-specific CTCF-binding sites might identify determinants of CTCF chromatin occupancy that are cell-type-invariant or erythroid-specific. We found that genetic variants disrupting CTCF motifs (1,459 discSNPs in 1,329 CTCF peaks) reveal both the positions and types of substitutions that are most poorly tolerated (Fig. 5a). The three regions most critical for binding (“CCA,” “AG,” and “GGC”) are also the most conserved nucleotides at high-occupancy CTCF-binding sites^54^ and contain the two nucleotides (2C, 12C) that when methylated are associated with decreased CTCF binding^53^. Notably, these data recapitulate in vitro data that 12C−>T significantly diminishes binding^54^ and additionally find several other mutations that are even more deleterious to in vivo CTCF binding.

We additionally identify several DNA sequences outside of the CTCF core motif that may positively or negatively regulate CTCF binding in erythroid cells (Fig. 5b). DiscSNPs in the CTCF motif itself have the strongest effects on CTCF binding, while those that disrupt other motifs, such as the CTCF-downstream motif identified by Nakahashi et al.^55^, exhibit milder effects, potentially due to constraints such as motif spacing. Notably, 4 of the 18 motifs that regulate CTCF binding correspond to the erythroid transcriptional regulators NFE2, TAL1, KLF1, and GFI1b. We hypothesized that these factors may be involved in directing erythroid-specific CTCF binding during differentiation. Intersection of CTCF peak sets from 23 mouse tissues identified CTCF peaks conserved in more than 60% of tissues (constitutive) and those found in fewer than 20% of tissues (variable). Of the variable peaks, we further identified those found specifically in erythroid tissues and those induced upon erythroid cell maturation (Supplementary Fig. 6a). Stratifying the effects of discSNP mutations to the CTCF motif by these groups reveals markedly stronger effects on CTCF binding at sites specific to differentiated erythroid cells than at constitutive peaks (Fig. 5c). In addition, erythroid-specific CTCF peaks are significantly enriched for both NFE2 (may promote CTCF binding) and GFI1b motifs (may reduce CTCF binding) relative to constitutive CTCF peaks (Fig. 5d). Together, these data suggest that tissue-specific CTCF sites are less robust to genetic variation and are sensitive to mutations within either the CTCF motif or proximal tissue-specific TF motifs.

### Influence of DNA motifs on erythroid chromatin accessibility

**Fig. 6:**
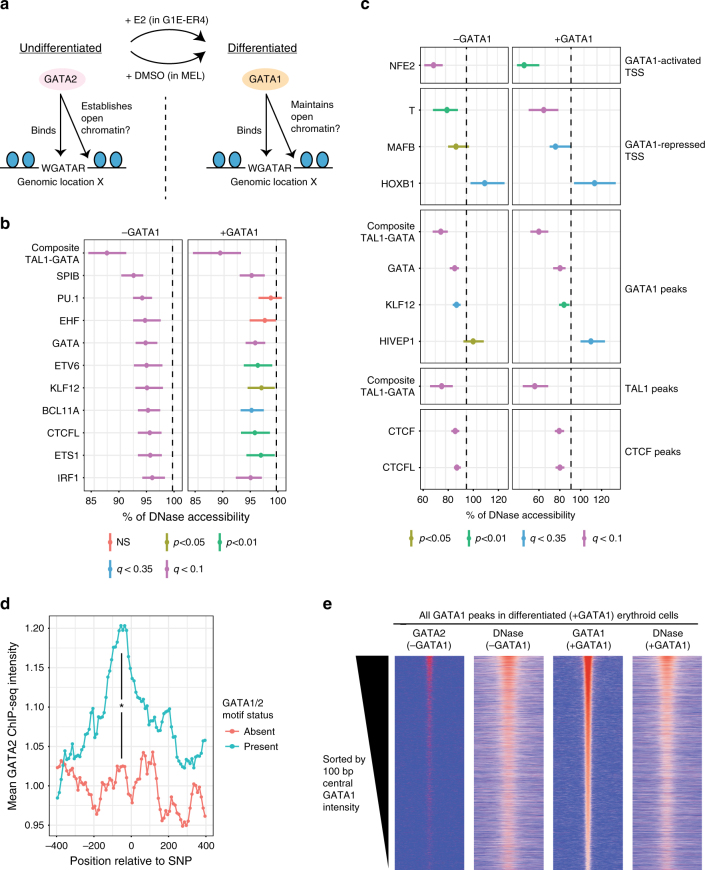
Genetic variation identifies DNA motifs that promote accessible chromatin at TF binding sites. **a** Model describing the undifferentiated GATA2^high^GATA1^low^ and the differentiated GATA2^low^GATA1^high^ erythroid states and the roles these TFs may play in regulating chromatin accessibility. **b** TFs whose motifs significantly alter genome-wide DNase peak signal (% impact, median ±  95% CI) when disrupted by a discSNP in undifferentiated (−GATA1) or differentiated (+GATA1) erythroid cells. Vertical line indicates no effect, and color indicates significance level. **c** TFs whose motifs significantly alter DNase signal (% impact, median ± 95% CI) at GATA1-regulated promoters or within TF binding peaks when disrupted by a discSNP in undifferentiated (−GATA1) or differentiated (+GATA1) erythroid cells. **d** Mean ChIP-seq intensity of GATA2 (in undifferentiated erythroid cells) at DNase peaks bound by GATA1 (in differentiated cells) at either an intact GATA motif or at one disrupted by a discSNP. Position is shown relative to the discSNP position (−400 bp upstream to 400 bp downstream). Significant differential binding (*) was assessed by BH-corrected Wilcoxon's test in the −200 to +200 region (*q* = 0.003). **e** Heatmaps showing the intensity of either GATA2 or GATA1 binding or DNase hypersensitivity in the undifferentiated (−GATA1) or differentiated (+GATA1) state at all sites that GATA1 binds in differentiated cells. Peaks (15,527) were sorted by GATA1 binding intensity in the differentiated state, regions span −1 kb to +1 kb centered on peak

To identify genetic determinants of chromatin accessibility we examined the impact of discSNPs on DNase hypersensitivity (DHS) in undifferentiated and differentiated erythroid cells (G1E-ER4 ± estradiol; MEL ± DMSO (dimethyl sulfoxide)). In agreement with previous reports^56–59^ we found that in spite of dramatic changes in gene expression during erythroid differentiation, genome-wide chromatin accessibility within DNase1 peaks is largely unchanged (Supplementary Fig. 7a), suggesting that genome accessibility is already mostly established in immature cells. Nevertheless, as the TF milieu changes during maturation it is possible that different factors are involved in the formation and maintenance of accessible chromatin sites. For example, in immature erythroid cells the major GATA binding protein GATA2 might play a role in DHS site formation^60^. In terminally differentiating cells, GATA1 replaces GATA2^30,61^ and might maintain chromatin accessibility or become dispensable for this function (Fig. 6a).

We examined in an unbiased manner the impact of discSNPs in all 726 TF motifs in the CIS-BP motif database^29^ on chromatin in both differentiation states. We found that composite GATA-TAL1 motifs have the strongest effects on chromatin accessibility genome-wide in immature erythroid cells (Fig. 6b), consistent with a requirement for GATA2 in DNase HS formation. In differentiated cells, GATA elements are also the most influential on DNase accessibility. However, it is impossible to distinguish whether this reflects the failure to establish accessible sites prior to maturation (the GATA2^hi^ GATA1^low^ state) or to maintain accessibility in the mature (GATA1^hi^ GATA2^low^) state. Less pronounced effects were seen with DNA motifs bound by other hematopoietic TFs, such as ETS binding sites (PU.1, EHF, ETV6, ETS1), Krüppel factor binding sites (KLF12), and BCL11A (Fig. 6b). It is important to bear in mind that motif identification does not definitively reveal the actual *trans*-acting factor bound to it.

Since GATA1-regulated genes typically have promoters that directly overlap DNase peaks, we exploited discSNPs within these overlapping regions to identify genetic determinants of chromatin accessibility at the promoters of GATA1-regulated genes. We found that mutations to NFE2 motifs significantly reduce TSS-proximal chromatin accessibility at genes whose transcription is activated by GATA1 (Fig. 6c). This finding is consistent with previous reports that NFE2 and GATA motifs are both required to establish accessibility at the β-globin locus control region^62,63^. Additionally, we found motifs that mediate either more (T Brachyury homolog, MAFB) or less (HOXB1) accessible chromatin at GATA1-repressed promoters (Fig. 6c). This finding suggests that GATA1-mediated repression involves a balance between factors that keep chromatin open, potentially to allow access by repressor complexes, and factors that ultimately promote a closed chromatin state.

We next examined the genetic requirements for chromatin accessibility specifically at GATA1, TAL1, and CTCF occupied sites. We found that the DNA motifs corresponding to each of these factors have significant roles in promoting open chromatin at their peak locations in both the undifferentiated and differentiated state (Fig. 6c). Additionally, for both GATA1 and CTCF, discSNPs within the TF peak impact TF occupancy and chromatin accessibility in a highly correlated fashion, and these correlations become stronger at more stringent FDR thresholds for differential TF binding (Supplementary Fig. 7b). Together, these data strongly implicate GATA1/2, TAL1, and CTCF as the relevant proteins that promote chromatin accessibility at their respective binding sites. Notably, the KLF12 (highly related to KLF1) motif (Supplementary Fig. 3a) also promotes open chromatin at GATA1 peaks, suggesting that chromatin accessibility at these locations may rely on collaborative TF binding (Fig. 6c).

The finding that GATA motifs promote accessibility at GATA1 peaks even in the undifferentiated state suggests that GATA2 may play a role in establishing open chromatin at these sites as mentioned above (Fig. 6d). In order to evaluate whether GATA2 may be establishing chromatin accessibility at sites that are later bound by GATA1 upon differentiation, we examined GATA2 binding in the undifferentiated state at these sites. In places where a GATA motif discSNP is associated with decreased accessibility, we found significant loss of GATA2 binding in the undifferentiated state (Fig. 6d). More broadly, levels of GATA2 and chromatin accessibility are correlated in the undifferentiated state (*r* = 0.55) at sites that are ultimately bound by GATA1 upon differentiation(Fig. 6e, Supplementary Fig. 7c). Higher GATA2 occupancy in the GATA2^hi^ GATA1^low^ undifferentiated state also correlates with chromatin features in differentiated cells such as accessibility (*r* = 0.50) and GATA1 occupancy (*r* = 0.48) (Fig. 6e, Supplementary Fig. 7d). Together, these data suggest that GATA2 establishes an open chromatin state at places later bound by GATA1 in differentiated erythroid cells. In sum, the analysis outlined here allows identification of DNA elements and in some cases the associated TF required for the establishment and/or maintenance of accessible chromatin.

## Discussion

Non-coding genetic variants impact TF binding, transcriptional regulation, phenotypic variation, and susceptibility to disease. Despite advances in GWAS fine-mapping^64^, high-throughput variant testing via reporter assays^65^, and gene editing, it remains a major challenge to connect exact genetic alterations to particular downstream phenotypes. In this study, we have established a framework for exploiting natural genetic variation between ENCODE cell lines to more precisely delineate the impact of genetic variants on TF binding and chromatin accessibility.

A prerequisite for our study is the identification of sequence variants in the genomes under investigation. Traditionally this has been accomplished by WGS. While a growing number of genome sequences exist for mouse strains^24^ and human cell lines^66^, WGS data are still lacking for many commonly used cell lines. The wide availability of ChIP-seq input data sets provided a readily available set of genomic sequences that could be mined to enable accurate identification of ~45% of variants within two murine erythroid cell lines and primary erythroid cells. We also found that the high numbers of background reads in ChIP-seq IP data sets yielded a rich resource for variant identification, provided that a wide range of IP types are used to avoid misclassification due to allelic imbalance. Other sources for genomic sequences (DNase-seq, ATAC-seq, Hi-C, etc.) should be similarly amenable to extraction of genetic variants for any given cell line.

We subsequently exploited DNA sequence variation between erythroid cell lines as an unbiased screen for the genetic determinants of TF binding and chromatin accessibility. Importantly, we demonstrated that mutations to the GATA1 motif have consistent effects on GATA1 binding regardless of which cell line contains the mutation. While we cannot exclude that distinct *trans* factor environments may subtly influence TF binding at a given site, the primary determinants of TF binding and chromatin accessibility defined here are encoded in the underlying DNA sequence. Natural variation that disrupts the canonical WGATAR motif affected in vivo GATA1 binding in ways not entirely expected from in vitro studies. For example, mutations of the core nucleotides are less detrimental (e.g., 29% binding reduction when G is mutated) to in vivo binding than expected, likely due to buffering by nearby contextual TF factors. Further, different substitutions at a given location are tolerated to varying degrees, potentially due to the impact of the variant nucleotide on local DNA shape^67^. Our analysis also discovered modulatory sequence variants that leave the GATA motif intact but fall into neighboring TF binding sites. Spacing of the sites relative to the GATA element was important, and in some cases inferences could be made as to the relevant *trans*-acting factor. Some motifs modulate GATA1 binding at unexpectedly large distances of >50 bp, suggesting collaborative binding that may be direct (short-range chromatin bending or a TF complex bridged by additional contextual TF factors) or indirect (e.g., via promoting accessible chromatin).

It is worth noting that studies aimed at examining collaborative TF binding often rely on global depletion of a TF, which can result in confounding secondary transcriptional effects. This drawback is avoided by the mining of *cis*-element variation as described here. Our results further support that in vivo TF binding is influenced in a variety of ways in addition to direct DNA contacts, which on the one hand might ensure robustness by buffering against the effects of binding site mutations, and on the other hand increase specificity through reliance on contextual TFs. Moreover, the contribution of neighboring DNA motifs to TF binding is consistent with previous findings that the binding profiles for a given TF can be dynamic across distinct cell lineages and differentiation states^52–54^. A striking example of such contextual effects is that motif alterations affect CTCF binding much less at constitutively occupied sites (6% median loss) when compared to sites at which binding is erythroid-specific (60% median loss) or differentiation stage dependent (80% median loss). These results highlight that even the chromatin occupancy of a factor as widely expressed as CTCF can be modulated in a highly context-dependent manner. It will be interesting to determine the contextual factors that convey the robustness of CTCF binding at constitutively occupied sites.

TFs can regulate chromatin occupancy of each other by modulating chromatin accessibility^4,68^. We identify DNA motifs corresponding to major erythroid TFs that promote chromatin accessibility in erythroid cells. While these data suggest that many TFs can impact chromatin accessibility, specific contexts, such as promoters and binding peaks for particular TFs, may rely on distinct subsets of these factors to establish and maintain accessibility. In particular, clear connections emerge between the regulation of TF binding and chromatin accessibility. For example, a KLF1-family DNA motif promotes both GATA1 chromatin occupancy and chromatin accessibility at GATA1-bound sites. Since the binding of GATA1 itself also promotes chromatin accessibility at GATA1 peaks, the effect of the KLF1 motif on accessibility may be direct, indirect (by promoting GATA1 binding), or a combination of the two. These data establish clear relationships between TF binding and chromatin accessibility and suggest that genetic variants can easily perturb both of these intertwined processes.

In sum the work outlined here provides evidence that genetic variation impacts in vivo TF binding and chromatin accessibility in ways that are not entirely predictable from in vitro studies and that depend highly on local chromatin context. Analyzing variants in a context-sensitive manner, as we and others^11–16^ have done, is critical to understanding and predicting the role of a particular variant in molecular phenotypes and phenotypic variation and disease. Our analysis framework is also readily adaptable to additional tissue types and chromatin features as well as to the analysis of new data sets from different mouse strains or patient samples that exhibit genetic variation. These analytic strategies may even be applicable in settings of somatic tumor mutations to identify alterations in TF binding that may impact cancer progression and metastasis. A limitation of our approach is that it compares quantitative information from ChIP-seq and DNase-seq experiments that may vary in design parameters (laboratory setting, read length, read depth, antibody lot, etc.). It is possible that applying our analytical approaches to experiments controlled for these parameters will provide even greater sensitivity at detecting contextual regulators of TF binding and chromatin accessibility. A relevant consideration here is that in our proof-of-concept experiments using a CRISPR-Cas9 guided mutagenesis followed by deep sequencing we were able to further define sequence requirements for GATA1 chromatin occupancy under conditions in which all the above variables can be controlled. We envision that this method can be extended to afford higher resolution and used for fine mapping of any DNA-binding protein or chromatin features such as accessibility and histone modifications.

Using genetic variation to more precisely define the functional components of TF binding sites as outlined here can enhance our mechanistic understanding of variation in inter-individual transcriptional output, molecular phenotypes, and common disease risk.

## Methods

### Evaluating similarity between cell lines

To evaluate similarity in transcriptional profiles, ENCODE tsv gene count files were obtained that corresponded to multiple replicates of RNA-seq data in differentiated G1E-ER4, MEL, and primary erythroblasts. After removal of ERCC spike-ins and non-ENSMUSG genes, genes were limited to those with minimum expected count of 35 in two replicates (reducing gene count from 30,000 to 12,000). We then used voom-limma^69^ for differential expression analysis between pairs of cell lines. This involved TMM normalization using edgeR (v3.16.5, default parameters) followed by limma/voom (v3.30.8, default parameters) for data fitting and determining differentially expressed genes.

To evaluate similarity in TF binding, aligned ChIP-seq reads and the associated peak calls were obtained from ENCODE. We used DiffBind^27^ (v3.6, parameters: minOverlap 1, summits 100, bRemoveDuplicates = T) to generate input-normalized and library-size-normalized binding intensities for each TF at the intersection of called peaks. We then computed Pearson's correlations between peak signal intensities. Pearson's correlation confidence intervals and differential comparisons are given based on Fisher’s Z-transform. For Fig. 1e, differential TF binding was defined as FDR (from DiffBind) < 0.05.

To evaluate similarity in DHS, aligned DNase-seq reads and the associated hotspot peak calls were obtained from ENCODE. Peak signal intensities were obtained at these peaks, library size normalized, and correlated between cell types using Pearson's correlation. For Fig. 1e, differential DNase peaks were defined as FDR (Benjamini–Hochberg corrected *t* test *p* values) < 0.05. For this analysis, data were grouped by hotspot size (since discSNP count/hotspot correlates with hotspot length) and the % of peaks with differential DNase signal was determined relative to peaks of that length containing no discSNPs.

These merged peak locations were later used as consensus locations for discSNP analysis.

### Calling genetic variation from input ChIP-seq data

Input ChIP-seq fastq files were obtained from ENCODE for each cell line analyzed. We used Trim Galore!^70^ (v0.4.1, parameters: phred33, -quality 20) to remove adapters and low-quality reads (ends < 20 bp; read length < 20 bp) and then bwa^71^ (v0.7.5a-r405, default parameters) to align these reads to the mm9 or hg19 genome builds. Picard (v1.121,default parameters; Picard Tools available http://broadinstitute.github.io/picard/) was applied to assign read groups and discard unmapped reads. We used multiple functions from GATK^22^ (v3.3, parameters:—variant_index_type LINEAR –variant_index_parameter 128000 –emitRefConfidence GVCF –T Haplotype Caller –genotyping_mode DISCOVERY—stand_emit_conf 10 –stand_call_conf 30) to identify variant nucleotide positions and the number of reference/variant reads found at these positions across all fastq files. We only considered variants with a total read depth > 5 and considered both read depth and variant read fraction in defining a homozygous variant nucleotide (Supplementary Table 1).

Following these assignments, DNA copy^72^ (v1.44.0, default parameters) was used to identify blocks of the genome containing at least ten variant calls where at least 90% of the calls share a zygosity of homozygous variant. Calls in these blocks that could not be assigned an accurate zygosity based on the procedures above (usually due to low read depth) were assigned the zygosity of their surrounding block.

For all comparisons of variants between erythroid cell lines, we considered only loci where one cell line contained a homozygous variant and a different cell line was homozygous reference.

### Benchmarking methods for calling genetic variation

Homozygous variants were called for the GM12878 (NA12878) cell line as described above. These variants were benchmarked against a set of gold standard homozygous variant calls identified by the Genome in a Bottle (GIAB) Consortium’s whole-genome sequencing of GM12878 cells^23^. Regions in the GIAB vcf file that fall within repeats or containing multiple alternative alleles were excluded from the gold standard set. This resulted in a gold standard set consisting of 1,099,780 SNPs and 136,109 indels. The homozygous reference gold standard calls are the 961,615 positions called by GATK as variant in at least one ChIP-seq dataset but not listed as variant in the GIAB vcf file. Evaluation of performance at lower starting read counts was performed by random downsampling of the 59 fastq files available for GM12878 into sets of either 50, 40, 30, 20, 15, 10, 8, 6, or 4 fastq files that were used as the input for our variant calling pipeline.

### TF motif calling

Motif scanning was completed by using the MEME suite^28^ fimo tool (v4.11.1, default parameters, uniform background) and CIS-BP motif database^29^ plus five additional motifs (GATA1-double, joint TAL1-GATA1, TAL1 alone, CTCF upstream/downstream). Motif hits for all GATA family motifs (Gata1/2/3/4/6) were merged as GATA1 motifs. The full set of motifs (726) was used for analysis of DNase data, while only motifs corresponding to TFs expressed in erythroid cells (175) were used for analysis of TF binding. For Fig. 5d, differential motif enrichment was performed using HOMER’s findMotifs tool^73^ (v4.9, default parameters) using the same motif database as above.

DiscSNPs that fell within TF binding peaks or DNase sites were tested for whether they disrupted a TF DNA motif as follows. A 40-bp window centered on the discSNP was scanned for motifs in order to find all motifs that contained the discSNP. The highest MEME score position for any given motif discSNP pair was determined and the MEME score for this motif in that exact position was collected for both alleles. For that discSNP pair, the highest MEME score was called the Score_max_ and the difference between MEME scores was called the DiffScore.

We used a random walk optimization approach to determine thresholds to call a motif as a true predicted binding site (Score_thres_) and to call a motif as disrupted by a discSNP (DiffScore_thres_). Values for these thresholds were randomly initialized and iteratively trained on all discSNPs that disrupt motifs for GATA1, TAL1, or CTCF. The impact of disruptions to these motifs on ChIP-seq binding intensity for their respective TF was optimized over 100 runs using 10× cross-validation. We found nearly optimal performance for each TF with Score_thres_ = 7.5 and DiffScore_thres_ = 2.5. We subsequently applied these thresholds to all motif scanning.

### Associating motif disruption with ChIP and DNase changes

DiscSNPs predicted to disrupt TF occupancy on a DNA motif were categorized by either TF motif, position within the motif, or identity of the variant nucleotide. ChIP-seq TF binding intensity was input-subtracted and library size normalized using DiffBind^27^. DNase intensity was library-size-normalized and signal distributions were quantile normalized. These normalized ChIP-seq and DNase-seq intensities were compared between the alleles contained the intact vs. disrupted motifs to identify the percent residual binding/accessibility intensity on the allele with motif disruption. Wilcoxon's tests were performed to determine if the percent residual intensity differed from 100%. Bootstrapping methods were used to generate 95% confidence intervals by randomly sampling with replacement for 1000 runs and median effect values for runs in the 2.5% and 97.5% percentiles. Multiple hypothesis testing between dependent tests was done by randomly permuting test group labels and using the *p* value of the most significant (smallest *p* value) Wilcoxon's test across 1000 runs to generate a null distribution, from which a *q* value reflects how many of these null *p* values are smaller than the actual test *p* value in question^74^. Identification of distance constraints on motif disruption (Fig. 4) was done by subsetting data into 10-bp sliding bins from a distance of 0 to 100 bp, overlapping by 5 bp. Multiple testing correction was done for these 20 bins for any given factor using permutation-based methods as described above.

### Prediction of GATA1 binding sites

Genome-wide GATA1 motif hits were determined by using the GATA1 position-specific weight matrix (PWM) and MEME’s fimo tool^28^ (v4.11.1, default parameters, uniform background). From these hits, a set of 27,272 positive GATA1 binding sites was constructed by filtering for hits in called GATA1 peaks (ENCFF001YFS). A set of 27,272 mono-nucleotide and di-nucleotide composition-matched negative GATA1 binding sites was constructed using MEME hits outside of GATA1 peaks for which normalized GATA1 IP read counts (ENCFF001MRW and ENCFF001MRR) are no more than the input-normalized read counts (ENCFF001MSP and ENCFF001MSK) inside a 400 bp window centered on the motif match. Read counts were extracted in motif match regions using BedTools multiBamCov^75^ (v2.240, default parameters) and normalized using mapped read totals overall 400 bp windows produced by multiBamCov. Positive and negative motif hits inside peaks containing discSNPs impacting a GATA1 motif were not used. MEME was then used to scan for contextual DNA motif regulators of GATA1 binding (considered all motifs that regulate GATA1 binding with Wilcoxon's *p* < 0.05, *n* = 25) within 100 bp of the GATA1 motif hit for both the positive and negative sets.

We first identified the contextual motifs that improved GATA1 binding site prediction in a logistic regression model that considers just the GATA1 MEME score vs. one that also considers the MEME score of a nearby contextual motif PWM. As a control, we evaluated the change in receiver operating characteristic curve area under the curve relative to addition of a random column-wise shuffle of the contextual motif PWM. We tested each motif against 100 random shuffles in 10 separate runs where the GATA1-positive binding set was compared to different random draws of GATA1-negative binding sets. Those motifs that outperformed their shuffles >90% of the time were then included in a logistic regression classifier that considered the MEME scores of the GATA1 motif and of each contextual motif. This regression model was evaluated relative to a model only containing the GATA1 motif MEME score and a separate model that includes random shuffles of the contextual motifs. All regression models were performed with 10× cross-validation to avoid overfitting.

Sensitivity of peak calling was compared between the GATA1 PWM only model and the model incorporating nearby contextual motifs by examining the true positive rate at an FDR of 0.3 (GATA PWM only model did not perform well enough to yield any threshold with an FDR of 0.1, so we relaxed the FDR to make a valid comparison).

### Cell culture

Culture of GATA1-containing erythroblast (G1E-ER4) cells has been described^21^. GATA1 was activated by the addition of 100 nM estradiol for 24 h. Culture of MEL cells has been described^76^. MEL cells were differentiated by the addition of 2% DMSO for 48 h. For ectopic retroviral integration experiments, both alleles of a TF binding site containing a discSNP were cloned into the MigR1 retroviral vector backbone. Retrovirus was packaged in 293T cells and used to infect G1E-ER4 cells. Cells were sorted for high GFP signal and qPCR was used to validate the copy number of the ectopic constructs as at least two alleles per cell. qPCR was performed with Power SYBR Green (Invitrogen).

ChIP was performed as previously described^40^. GATA1 N6 antibody sc-265 (Santa Cruz) and rat serum immunoglobulin G I8015 (Sigma-Aldrich) were used at 10 μg/IP reaction. qPCR primers are listed in Supplementary Table 2 for both endogenous sites and ectopic site qPCR barcodes.

For RNA qRT-PCR, we isolated RNA using TRizol (Life Technologies). Reverse transcriptase reaction was performed using iScript (Bio-Rad). qPCR primers are listed in Supplementary Table 2. Transcript quantifications are normalized to a panel of reference genes (*B-actin*, *Gapdh*, *Fog1*, *Gata2*, *Klf1*, *Pabpc1*, *Slc4a1*, *Spna1*) using median CT value in order to reduce clonal variability.

Targeted editing of the *Bola1*-proximal GATA1 peak was conducted in Cas9 + MEL cells. The Cas9 expression vector was derived by inserting the *Streptococcus pyogenes* Cas9 CDS (Addgene: #49535) into a lentiviral EFS-Cas9-P2A-Puro expression vector. This Cas9 lentiviral vector was termed LentiV_Cas9_puro. The MEL cells were lentiviral transduced with LentiV_Cas9_puro and selected with 5 μg/ml puromycin for 5 days. These MEL-Cas9 cells were then infected with a Lenti-gRNA-GFP (LRG, Addgene: #65656) vector containing either the targeting *Bola1* gRNA or a control gRNA targeting a GATA1 peak in the *AW011738* gene. To generate clones containing bi-allelic deletions, GFP-positive cells were sorted into 96-well plates and screened by genomic DNA PCR and Sanger Sequencing. High-throughput screening of GATA1 binding required collecting GFP-positive cells in bulk and performing GATA1 ChIP using this cell population.

### Targeted GATA1 ChIP-seq at a Cas9-edited locus

Deep sequencing libraries of the GATA-bound *Bola1* promoter region were constructed using a previously published strategy^77^. The *Bola1* locus from ChIP input and GATA1 IP material was PCR amplified such that the wild-type sequence would yield a 186 bp target amplicon. The PCR product was end-repaired using T4 DNA polymerase (NEB), DNA polymerase I, large (Klenow) fragment (NEB), and T4 polynucleotide kinase (NEB). An A-overhang was then added to 3′ end using Klenow fragment (3′−5′ exo-) (NEB). Finally, the DNA fragment was ligated with custom barcodes, and PCR-amplified with Illumina paired-end sequencing primers^77^. Library size was determined (average ~470 bp) using the Agilent Bioanalyzer 2100, followed by quantitation using real-time PCR using the KAPA Library Quant Kit for Illumina (KAPA Biosystems catalog no. KK4835). Paired-end sequenced (2 × 150 bp) was performed on the Illumina NextSeq 500 in the high output mode using Illumina sequencing reagents according to the manufacturer’s instructions, and bcl2fastq2 v2.15.0.4 was used to convert the reads to fastq using default parameters. Read pairs were merged into a single sequence using Flash^78^ (v1.2.11, parameters: -M 125 –m 4) and aligned to the wild-type sequence using Needle^79^ (v6.6.0, parameters: -gapopen = 10 -gapextend = 0.5 -awidth3 = 5000). Each sample had between 68 and 76 million read pairs with >80% identity to the wild-type sequence that either matched the wild-type sequence or had a single deletion. Read counts for a particular deletion-containing allele were normalized by the wild-type allele read counts for that replicate such that the wild-type allele enrichment ratio (IP read count/Input read count) = 1. For Fig. 3d, enrichment ratios for 1-bp or 2-bp deletions were aggregated into sliding windows and tested for enrichment <1 using Wilcoxon's tests.

### Data availability

The data sets generated during and/or analyzed during the current study are available from the corresponding author upon reasonable request.

## Electronic supplementary material

Supplementary Information

Descriptions of Additional Supplementary File

Supplementary Data 1
