# Supplementary material for: Exploiting genetic variation to uncover rules of transcription factor binding and chromatin accessibility
Source: Nat Commun. 2018 Feb 22;9:782. doi: 10.1038/s41467-018-03082-6 (PMC5823854; doi:10.1038/s41467-018-03082-6)
Supplement: Supplementary file 1 — Supplementary Information [file 41467_2018_3082_MOESM1_ESM.pdf]

Supplementary Figure 1

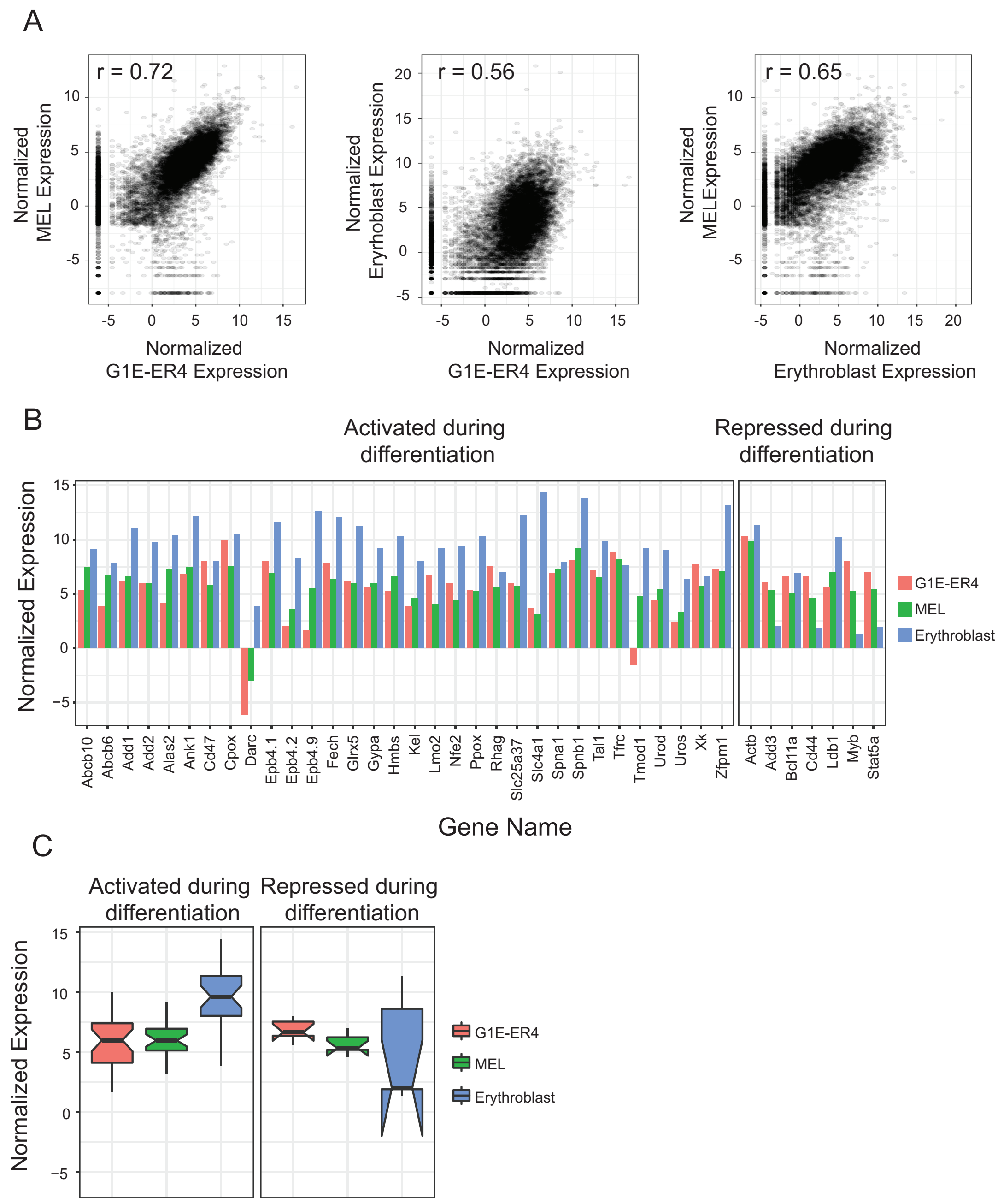

,

Supplementary Figure 1: Comparison of murine erythroid cell lines**. a**) DESeq2-normalized gene counts in G1E-ER4, MEL, or Erythroblast are plotted against each other, Spearman Correlation Coefficient listed. (**b**) Normalized gene counts in G1E-ER4, MEL, or Erythroblast for a panel of genes that are activated or repressed during erythroid differentiation[18]. (**c**) Aggregated gene counts for activated/repressed genes. Boxplot center is median, hinges are 25% and 75% percentiles, whisker is no greater than 1.5*IQR (Inter-quartile range).

Supplementary Figure 2

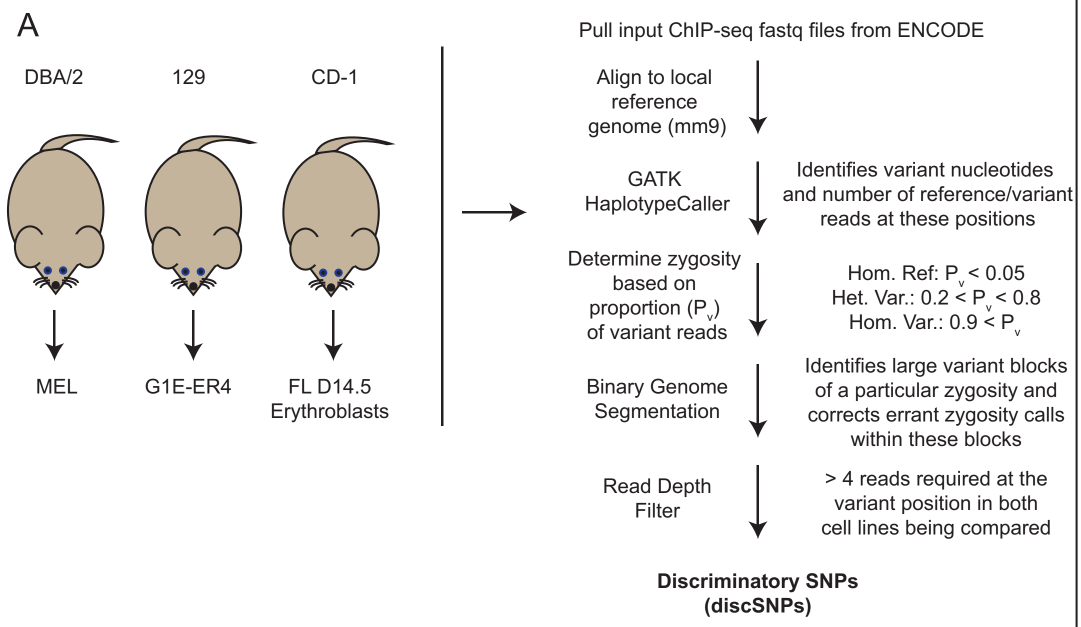

B

Type	Erythroblast	G1E−ER4	MEL
ChIP total	1729338 (100%)	2593064 (47%)	2224247 (41%)
Sanger total	0 (0%)	5169248 (94%)	5180142 (96%)
ChIP unique	1729338 (100%)	356762 (6%)	209413 (4%)
Sanger unique	0 (0%)	2932940 (53%)	3165304 (59%)
ChIP/Sanger Intersection	0 (0%)	2236302 (40%)	2014834 (37%)
ChIP/Sanger Union	1729338 (100%)	5525926 (100%)	5389537 (100%)

,

<u>Supplementary Figure 2</u>: Pipeline for variant calling from ChIP-seq data. (**a**) Schematic outlining methods for identifying genetic variation between murine erythroid ENCODE cell lines. The cell lines are listed with their strains of origin. Input ChIP-seq files corresponding to each of these cell lines are mapped to the reference mouse mm9 genome. The GATK HaplotypeCaller tool identifies variant nucleotide locations and the number of high-quality reads corresponding to reference or variant sequence at these locations. Zygosity is re-assigned based on stringent criteria based on proportion of variant read count. Binary genome segmentation identifies blocks of a particular variant zygosity and corrects variants of ambiguous zygosity within these blocks. Finally, a read depth filter is applied to any genome variants in order to identify high-confidence calls. These calls represent homozygous variants relative to the mm9 genome. Comparison of variant nucleotides between cell lines identifies the set of discriminatory SNPs (discSNPs) that discriminate any pair of erythroid cell lines. (**b**) Table summarizing the variants identified by either ChIP-seq on the cell line of interest or from the Sanger institute's characterization of the parent mouse strains.

Supplementary Figure 3

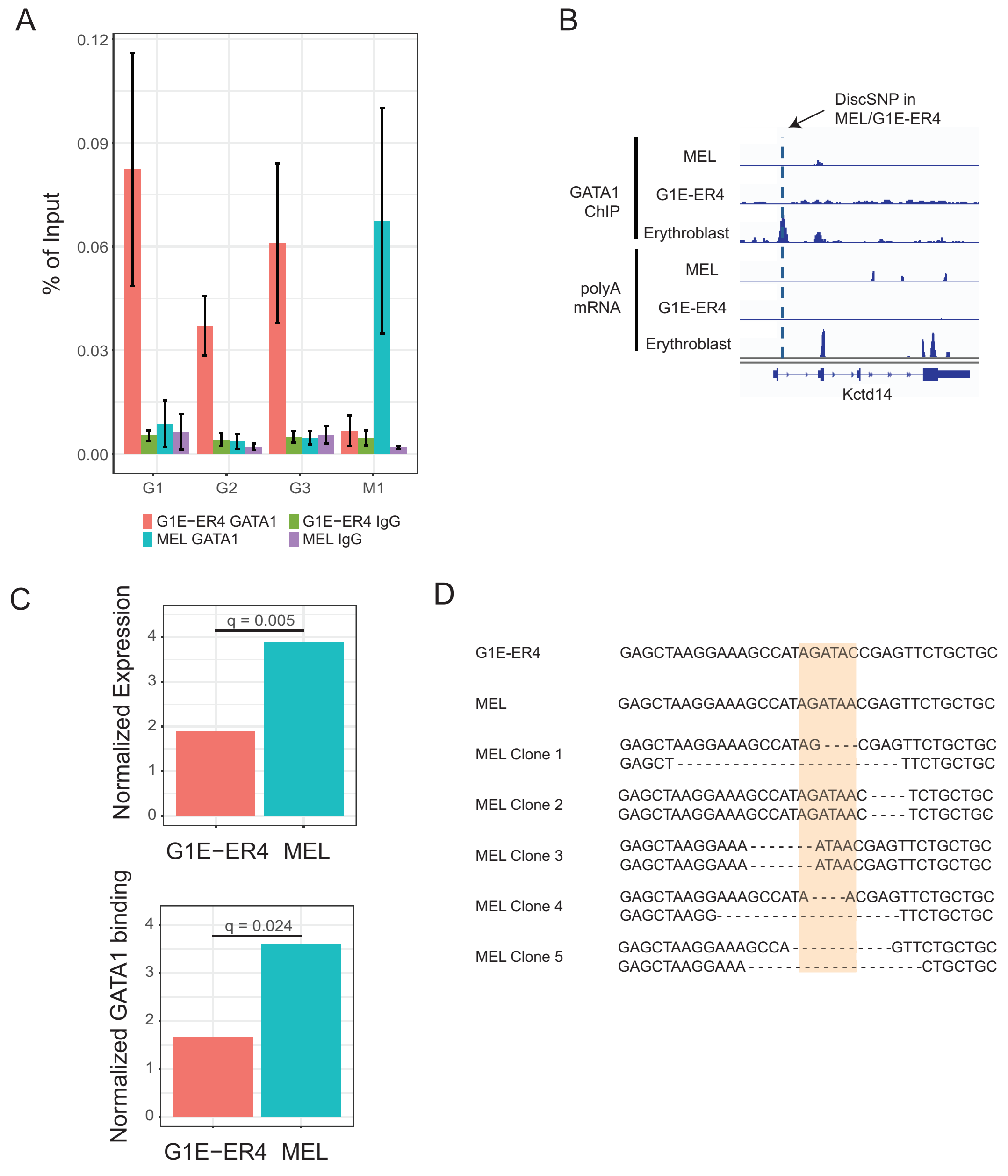

,

<u>Supplementary Figure 3</u>: Single nucleotide variants are associated with large changes in GATA1 binding and local transcription. (**a**) GATA1 ChIP-qPCR in differentiated G1E-ER4 (24h E2) and differentiated MEL (48h DMSO) at sites containing a single discSNP. The discSNP is associated with binding in G1E-ER4 only (G1 [chr15: 31,471,995 – 31,472,087], G2 [chr3: 83,831,768 - 83,831,850], G3 [chr6: 134,784,747 – 134,784,808]) or in MEL only (M1 [chr12:40,912,197 – 40,912,282]). Mean +/- SEM, n= 3. (**b**) GATA1 ChIP-seq and RNA-seq intensity tracks (input and library size normalized, identical y-axis scales) reveals dramatic changes to both GATA1 binding and Kctd14 transcription in MEL and G1E-ER4 cells, which contain a single nucleotide change at the dotted vertical line (no other discSNPs within 1kb). (**c**) Differential expression of Bola1 and GATA1 binding to the Bola1-proximal GATA1 peak seen in G1E-ER4 and MEL cells. Expression and binding q-values are Benjamini-Hochberg adjusted using DESeq2. (**d**) Sequence of the Bola1-proximal GATA1 peak in wild type G1E-ER4, wild type MEL, or the 5 MEL clones containing bi-allelic deletions. Canonical GATA1 binding sequence (WGATAR) is highlighted.

Supplementary Figure 4

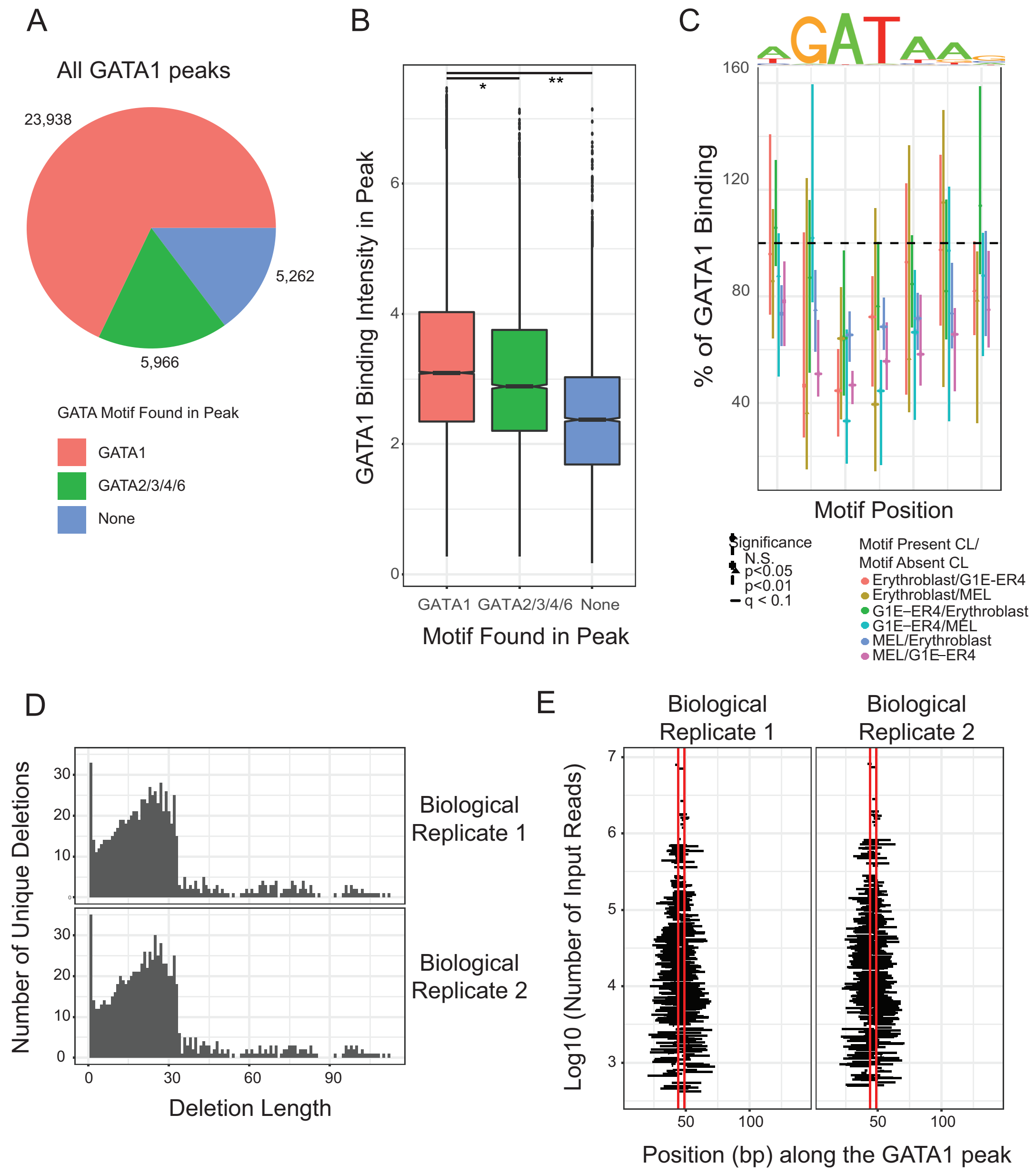

,

Supplementary Figure 4: Dissecting single nucleotide determinants of GATA1 binding. (**a**) The majority of GATA1 peaks have a GATA1 motif, while about half of the remaining peaks have a motif for a different GATA1 factor and the rest have no GATA motif at all. (**b**) GATA1 binding intensities stratified by whether the peak contains a GATA1, a GATA2/3/4/6, or no GATA-family motif. Wilcoxon test: *p = 4e-30, **p=4e-162. Boxplot center is median, hinges are 25% and 75% percentiles, whisker is no greater than 1.5*IQR. (**c**) The percent impact (median +/- 95% CI) on GATA1 binding intensity associated with mutations at various positions when stratified by the cell line with the intact GATA1 motif and the cell line with the disrupted GATA1 motif. Data sparsity at some positions prevented all possible pairwise comparisons. (**d**) Histogram of deletion length generated by targeting Cas9 to the Bola1-proximal GATA1 peak. (**e**) Positions of deletions relative to the WGATAR motif (red lines) plotted vs deletion frequency in sequenced ChIP Input material.

Supplementary Figure 5

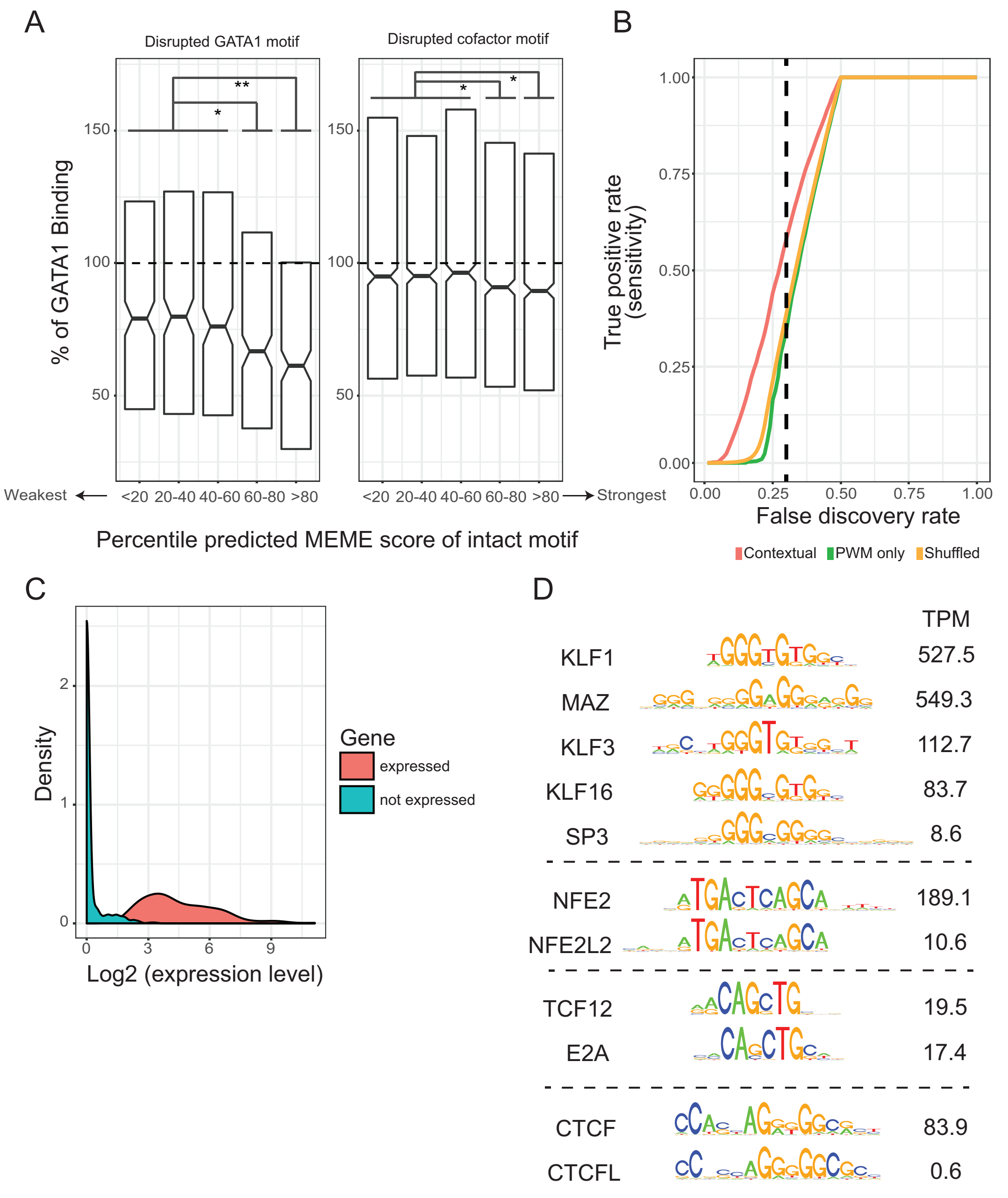

<u>Supplementary Figure 5:</u> Contextual motifs impact GATA1 binding**. (a)** DiscSNPs that directly disrupt a GATA/non-GATA motif have variable impacts on GATA1 binding depending on the predicted MEME score of the intact motif. Wilcoxon, * $p < 0.01$, ** $p = 1.8e-7$. Boxplot center is median, hinges are 25% and 75% percentiles. (**b**) Sensitivity of identification of GATA1 binding peaks at an FDR of 0.3 (dotted line) when either using the GATA PWM alone, the GATA1 PWM + contextual motif PWMs, or control shuffles of contextual motif PWMs. (**c**) Density plot of log2-scale RNA-seq expression values for TFs corresponding to all TF DNA motifs in the Cis-BP motif database in differentiated erythroid cells (G1E-ER4). (**d)** Transcription factor motifs highly related to KLF1, NFE2, TCF12, and CTCF are depicted along with expression in transcripts per million (TPM) of the corresponding factor in differentiated G1E-ER4 cells (24h E2).

Supplementary Figure 6

A

All CTCF peaks (across 23 tissues)	150261
Constitutive binding peaks (>60% of tissues)	21886
Variable binding peaks (<20% of tissues)	106125
Erythroid−specific variable peaks	27120
Erythroid specific peaks induced upon terminal differentiation	2824

Supplementary Figure 6: Tissue conservation of CTCF binding sites. (**a**) Intersection of CTCF bed files between 23 tissues identifies peaks found in > 60% of tissues, those found in <20%, those found in <20% where at least 1 tissue is an erythroid cell line, and the subset of these erythroid-specific peaks that are present in differentiated G1E-ER4 cells but not in undifferentiated G1E cells.

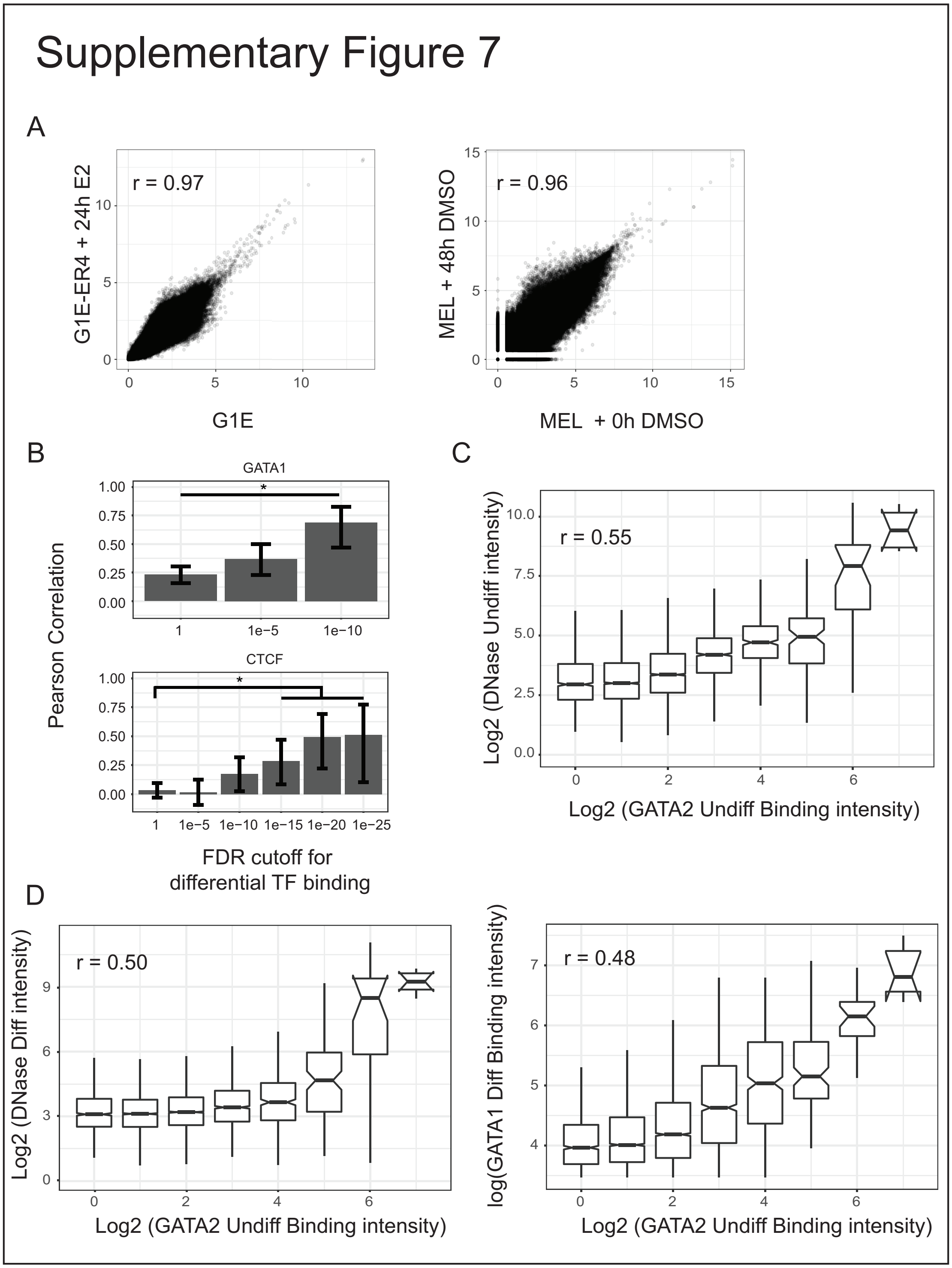
Supplementary Figure 7
A
r = 0.97
G1E-ER4 + 24h E2
G1E
r = 0.96
MEL + 48h DMSO
MEL + 0h DMSO
B
GATA1
CTCF
Pearson Correlation
FDR cutoff for
differential TF binding
C
r = 0.55
Log2 (DNase Undiff intensity)
Log2 (GATA2 Undiff Binding intensity)
D
r = 0.50
Log2 (DNase Diff intensity)
Log2 (GATA2 Undiff Binding intensity)
r = 0.48
log(GATA1 Diff Binding intensity)
Log2 (GATA2 Undiff Binding intensity)

Supplementary Figure 7: Sequence determinants of chromatin accessibility. (**a**) Log-log plot between DNase signal in an undifferentiated state vs differentiated state for either G1E/G1E-ER4 or MEL. Pearson correlation coefficient is shown on the plot. (**b**) For discSNPs disrupting either a GATA1 or CTCF motif within GATA1 and CTCF peaks, respectively, Pearson correlation coefficients between differential chromatin occupancy and differential DNase1 accessibility at a range of FDR cutoffs for differential binding. Bars are 95% confidence intervals, * $p < 0.05$ (Fisher's z-transform). (**c**) Log-log plot between GATA2 binding in undifferentiated cells and DNase intensity in undifferentiated cells, or with (**d**) DNase intensity in differentiated cells (left) or GATA1 intensity in differentiated cells (right). For (**c**) – (**d**), binding is examined at 15,527 GATA1 peaks called in differentiated erythroid cells. Pearson correlation coefficient is shown on the plot. Boxplot center is median, hinges are 25% and 75% percentiles, whisker is no greater than 1.5*IQR.

Supplementary Table 1: Variant calling read thresholds

1) Total read depth < 9 AND no variant reads or<br>2) Total read depth >= 11 AND variant read fraction < 0.05	Homozygous reference
1) Total read depth >= 11 AND variant read fraction > 0.9	Homozygous variant

Supplementary Table 2: qPCR primer sequences

Experiment	Location	Type	Left Primer	Right Primer
SuppFig3b_Endogenous GATA1 ChIP	G1	endogenous	ctctgtccccagaaacaatg	tttacactccactcctctgagc
SuppFig3b_Endogenous GATA1 ChIP	G2	endogenous	ttgctgctgctatctgagaa	ggaatgcactatttgaaaactctc
SuppFig3b_Endogenous GATA1 ChIP	G3	endogenous	gaagccaaaccatgtccaac	atactgactgcgtgtgcttctc
SuppFig3b_Endogenous GATA1 ChIP	M1	endogenous	cagagccaagtgtgtggcta	tagaaatggggaagccaagg
Fig2c_Ectopic GATA1 site testing	Panel 1 (AGATAA -> AGATAG)	endogenous	aaggcagtgacctgctttct	ttcgatcatttccccttgat
Fig2c_Ectopic GATA1 site testing	Panel 2 (AGATAG -> AGATGG, same site as G3 above)	endogenous	gaagccaaaccatgtccaac	atactgactgcgtgtgcttctc
Fig2c_Ectopic GATA1 site testing	Panel 3 (AGATAA -> AGATGA)	endogenous	gctctggtcttacgggaaca	cctaggctgaccttgtgacc
Fig2c_Ectopic GATA1 site testing	Panel 4 (AGATAA -> AGAAAA, same site as G1 above)	endogenous	ctctgtccccagaaacaatg	tttacactccactcctctgagc
Fig2c_Ectopic GATA1 site testing	barcode_reference_allele	ectopic	gacctgcagttcgaagttcct	cataagaacaagcttcttaacaac
Fig2c_Ectopic GATA1 site testing	barcode_variant_allele	ectopic	gacctgcagttcgaagttcct	cagcctgtcgataaccacgttagt
Fig2d_GATA1 Bola1 occupancy	IVR16	endogenous	tggccatttttactatgttaattttgc	tagacttgtcatggttatggattgg
Fig2d_GATA1 Bola1 occupancy	HS2	endogenous	gggtgtgtggccagatgttt	caccttccctgtggacttcct
Fig2d_GATA1 Bola1 occupancy	Bola1	endogenous	agctcatggccaaaatgg	atctccaaactgctccagctc

Fig2d_Bola1 RNA qRT-PCR	Bola1	endogenous	gaccggaaattcg gttgttag	cgcacttagcattc gctttg
Fig2d_Bola1 RNA qRT-PCR	B-actin	endogenous	acacccgccacc agttc	tacagcccgggg agcat
Fig2d_Bola1 RNA qRT-PCR	Gapdh	endogenous	aggttgtctcctgc gacttca	ccaggaaatgag cttgacaaag
Fig2d_Bola1 RNA qRT-PCR	Fog1	endogenous	gtcagggtgaaga cagaactg	tgtggcagaaag agtgtcc
Fig2d_Bola1 RNA qRT-PCR	Gata2	endogenous	cacccctaagcag agaagcaa	tggcaccacagtt gacacact
Fig2d_Bola1 RNA qRT-PCR	Klf1	endogenous	cacgcacacggg agagaag	cgtcagttcgtctg agcgag
Fig2d_Bola1 RNA qRT-PCR	Pabpc1	endogenous	ctaccagccagca cctcctt	tgcagcacggttct gagtct
Fig2d_Bola1 RNA qRT-PCR	Slc4a1	endogenous	tggaggcctgatc cgtgata	agcgcatcggtga tgtca
Fig2d_Bola1 RNA qRT-PCR	Spna1	endogenous	aaagagttccgct cttgcctgaga	tttcctccctggatc cacagcatt
Fig2d_BolaCas9 Targeting_gDNA_screening	Bola1	endogenous	tgtgtgagggaga acacagc	gcaagtggatcttt gggttc
Fig2d_BolaCas9 Targeting_gRNA	Bola1	endogenous	caccgcagcaga actcgttatcta	aaactagataacg agttctgctgc
Fig2d_BolaCas9 Targeting_controlgRNA	AW011738	endogenous	caccgttcttggctg aacttagcgg	aaacccgctaagt tcagccaagaac
Fig3c_Bola1NextSeq	Bola1	endogenous	agaagctggaga agggaagg	caaagacaaggc tgctgttg
