# Supplementary material for: Exploiting genetic variation to uncover rules of transcription factor binding and chromatin accessibility
Source: Nat Commun. 2018 Feb 22;9:782. doi: 10.1038/s41467-018-03082-6 (PMC5823854; doi:10.1038/s41467-018-03082-6)
Supplement: Supplementary file 2 — Descriptions of Additional Supplementary File [file 41467_2018_3082_MOESM2_ESM.pdf]

Description of Supplementary Files

File Name: Supplementary Data 1
Description: ENCODE ChIP-seq datasets used for variant calling and binding analysis.
